# Silicon Photonic Biosensors Using Label-Free Detection

**DOI:** 10.3390/s18103519

**Published:** 2018-10-18

**Authors:** Enxiao Luan, Hossam Shoman, Daniel M. Ratner, Karen C. Cheung, Lukas Chrostowski

**Affiliations:** 1 Department of Electrical and Computer Engineering, University of British Columbia, 2329 West Mall, Vancouver, BC V6T 1Z4, Canada; hoshoman@ece.ubc.ca (H.S.); kcheung@ece.ubc.ca (K.C.C.); lukasc@ece.ubc.ca (L.C.); 2 Department of Bioengineering, University of Washington, 3720 15th Ave. NE, Seattle, WA 98195-5061, USA; dratner@uw.edu

**Keywords:** silicon photonics, evanescent optical field sensor, label-free SOI biosensor, Mach–Zehnder interferometer, ring resonator, photonic crystal, Bragg grating, sub-wavelength grating, lab-on-a-chip, microfluidics

## Abstract

Thanks to advanced semiconductor microfabrication technology, chip-scale integration and miniaturization of lab-on-a-chip components, silicon-based optical biosensors have made significant progress for the purpose of point-of-care diagnosis. In this review, we provide an overview of the state-of-the-art in evanescent field biosensing technologies including interferometer, microcavity, photonic crystal, and Bragg grating waveguide-based sensors. Their sensing mechanisms and sensor performances, as well as real biomarkers for label-free detection, are exhibited and compared. We also review the development of chip-level integration for lab-on-a-chip photonic sensing platforms, which consist of the optical sensing device, flow delivery system, optical input and readout equipment. At last, some advanced system-level complementary metal-oxide semiconductor (CMOS) chip packaging examples are presented, indicating the commercialization potential for the low cost, high yield, portable biosensing platform leveraging CMOS processes.

## 1. Introduction

Medical diagnostics have come to play a critical role in healthcare by providing early detection and diagnosis of disease [1], improving timely and appropriate care [2], protecting the safety of medical products such as blood for transfusion [3], and reducing healthcare costs [4]. Most diagnostic systems have been designed to meet the requirements of well-funded clinical laboratories in highly regulated environments, but do not address the need of the majority of patients and caretakers in the developing world with inadequate healthcare facilities and clinical laboratories [5]. For instance, the enzyme-linked immunosorbent assay (ELISA), which has been the gold-standard method in biomarker detection and validated for more than 40 years, can obtain an ultra-low detection limit (∼1 pM) [6]. However, this method is based on a label-based approach which delays results, adds to costs due to specialized reagent requirements, and needs complex micro-evaluations using large, automated analyzers. Therefore, highly sensitive, fast and economic techniques of analysis are desired for both developing and developed countries for point-of-care (POC) diagnostic applications to improve access to cost-effective healthcare technologies.

The development of practical biosensors is one of the most promising approaches to satisfy the growing demand for effective medical diagnostic technologies [7]. Since the first oxygen electrode biosensor demonstrated by Clark in 1956 [8], scientists and engineers have made significant progress in the field of biosensing techniques, which has subsequently been adopted into clinical practice. By 2020, the global biosensors market size is anticipated to reach USD 21.17 billion, among which optical biosensors are identified as the most lucrative technology segment [9]. This represents just a fraction of the estimated USD 72 billion worldwide markets for in vitro diagnostics (IVD). There are a variety of techniques that have been successfully employed for optical measurements, such as emission, absorption, fluorescence, refractometry, and polarimetry [10]. Evanescent field detection is the primary detection principle of many optical biosensors [10]. Due to the sensitivity to changes in the local refractive index (RI) within the evanescent field surrounding the device, evanescent field biosensors such as Surface Plasmon Resonance (SPR) or planar waveguide based sensors have attracted growing interest for sensitive, real-time, and label-free biomolecular detection [11]. Wavelength (or phase) interrogation and intensity interrogation are two common interrogation configurations applied among these transducers.

Several technologies are available for the fabrication of photonic biosensors, and the well-developed silicon photonic integrated circuits (PICs) technology is one of the most promising [12]. Due to the compatibility with complementary metal-oxide semiconductor (CMOS) foundry processes, silicon PICs can be manufactured with great efficiency at high volume [13]. Moreover, the high refractive index contrast between silicon and silicon dioxide, or other surrounding media, enables the development of miniaturized compact sensing devices, with the additional possibility of fabricating multiple sensors on one single chip [10]. Meanwhile, silicon photonics are excellent transducers for continuous and quantitative label-free biosensing [14,15], which can directly respond to affinity interactions between analyte and receptor molecules in real-time. Hence, numerous silicon photonic sensing devices, such as Mach–Zehnder interferometers (MZIs) [16,17], microring resonators (MRRs) [18,19], microdisk resonators [20,21], Bragg grating resonators [22,23], and one-dimensional (1D) or two-dimensional (2D) photonic crystals (PhCs) [24,25] have been developed over the past decades for biosensing diagnostic applications.

This paper reviews the literature on label-free integrated (i.e., not SPR) photonic biosensors over the last 20 years. An overview of the main planar integrated optical sensing configurations for label-free detection is presented, emphasizing the description of these structures and corresponding sensing mechanisms. Several performance-improving approaches, such as using slot, thinner or suspended waveguides, and 1.31 µm wavelength light sources, as well as advanced strategies by employing sub-wavelength grating (SWG) waveguides and the Vernier effect method, are also introduced. A brief summary of experimental validations of biomarkers and their respective detection limits (DLs) is listed to illustrate their dynamic ranges of sensing and limitations therein. To address system operations for lab-on-a-chip diagnosis, approaches for optofluidic and optoelectronic integrations on the silicon-on-insulator (SOI) substrate are mentioned including their advantages and disadvantages. Finally, examples of some state-of-the-art packaged on-CMOS sensing platforms are reported, showing a promising prospect for the development of fully integrated, portable, lab-on-a-chip biosensing architectures for multiplexed label-free diagnostics.

## 2. Theory and Structures

### 2.1. Evanescent Field Sensing Principle

**Figure 1:**
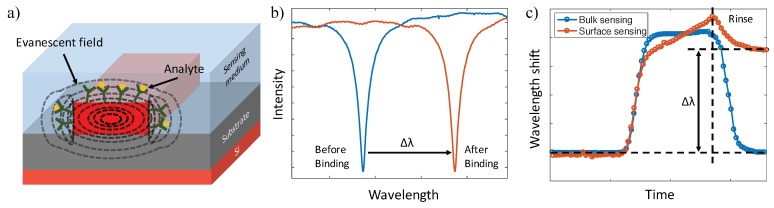
Principle of the evanescent field detection for a silicon photonic biosensor. (**a**) The evanescent field (dashed lines) around the waveguide is sensitive to the RI change caused by biological binding events at the waveguide’s surface. (**b**) Optical transmission spectra of the sensor before (blue curve) and after (red curve) the analyte interaction, resulting in a wavelength shift (Δλ). (**c**) Sensorgrams of the sensor in bulk (blue curve) and surface (red curve), where the signals are recorded as a function of time.

Leveraging the SOI platform, silicon photonic biosensors rely on near-infrared light confined in nanometer-scale silicon wires (known as waveguides) to sense molecular interaction events. The portion of the light’s electrical field traveling outside of the waveguide is referred to as the evanescent field, which can interact with the surrounding volume to create an external RI sensitive region (Figure 1a). When target molecules bind to receptors at the waveguide’s surface, the accumulation of molecules with a different refractive index changes the external RI and perturbs the evanescent field, which then further influences the behavior of the guided light in the waveguide [26]. By monitoring the coupling and/or propagation properties of the output light, analytes of interest can be detected in real-time (Figure 1b) [27]. Since the evanescent field decays exponentially with a decay length ranging from a few tens to a few hundreds of nanometers into the bulk medium, the sensing signal of an analyte captured within the decay length shows a significant difference compared to the signal of an analyte floating far away from the surface [15]. Thus, based on the response of the evanescent field sensor, we can distinguish the target molecules immobilized on the surface (surface sensing) from those remaining in bulk solution (bulk sensing), as presented in Figure 1c.

Several figures of merit are widely used for the evaluation of sensor performance, such as selectivity, reproducibility, stability, sensitivity, and resolution (detection limit). Selectivity describes the ability of a sensor to detect a target analyte in a sample containing other admixtures, which is the main consideration for the bioreceptor selection; reproducibility is the ability to generate identical responses for repetitive experimental setups, which provides high reliability and robustness for the signal; and stability refers to the degree of susceptibility to ambient disturbances around the sensing system, which can affect the precision and accuracy of the sensor [28]. Sensitivity (S) and DL are the two performance criteria we focus on in this review since they have stronger correlation with their sensor geometries. In evanescent field sensors, sensitivity is determined by the strength of interactions between matter and the fraction of light in solution or at the surface [15]. According to the status of target molecules, two specific types of sensitivities are defined in biosensing applications: (i) bulk sensitivity (S_bulk_), which takes into account RI changes of the waveguide’s entire cladding; and (ii) surface sensitivity (S_surf_), which assesses RI changes within the first few tens to hundreds of nanometers above the surface [26]. For the bulk sensitivity, it is defined as the slope of wavelength (or phase) shift versus the change of refractive index unit (RIU), and the shift is described by Chrostowski et al. [29]:

(1) $$\frac{\Delta \lambda }{\lambda }\;(\mathrm{or})\;\frac{\Delta \phi }{\phi }\;=\;K\times \frac{\Delta n_{\mathrm{fluid}}}{n_{g}}\left(\frac{\partial n_{\mathrm{eff}}}{\partial n_{\mathrm{fluid}}}\right),$$

where λ is the wavelength, ϕ is the phase of the input light, *K* is the sensor structure constant (varies depending on the configuration of the sensor), $n_{\mathrm{fluid}}$ is the RI of the analyte solution, and $n_{\mathrm{eff}}$ and $n_{g}$ are the mode’s effective and group indices. From Equation (1), the wavelength (or phase) shift is mainly contributed by the shift in the solution’s RI ($\Delta n_{\mathrm{fluid}}$), the dispersion ($n_{g}$) of the material and waveguide, and the mode’s effective index change ($\partial n_{\mathrm{eff}}$/$\partial n_{\mathrm{fluid}}$) caused by the slight change of the mode profile [29]. The bulk sensitivity is defined as:

(2) $$S_{\mathrm{bulk}}\;=\;\frac{\Delta \lambda \;(\mathrm{or})\;\Delta \phi }{\Delta n_{\mathrm{fluid}}}.$$

As for the surface sensitivity, the definition is slightly different from the bulk one by replacing the solution’s RI ($n_{\mathrm{fluid}}$) with the thickness of a homogeneous adlayer on the surface ($t_{\mathrm{adlayer}}$). Therefore, the expressions for the wavelength (or phase) shift and surface sensitivity are:

(3) $$\frac{\Delta \lambda }{\lambda }\;(\mathrm{or})\;\frac{\Delta \phi }{\phi }\;=\;K\times \frac{\Delta t_{\mathrm{adlayer}}}{n_{g}}\left(\frac{\partial n_{\mathrm{eff}}}{\partial t_{\mathrm{adlayer}}}\right),$$

and

(4) $$S_{\mathrm{surf}}\;=\;\frac{\Delta \lambda \;(\mathrm{or})\;\Delta \phi }{\Delta t_{\mathrm{adlayer}}},$$

respectively [30]. From Equations (3) and (4), $\partial n_{\mathrm{eff}}$/$\partial t_{\mathrm{adlayer}}$ is highly dependent on the refractive index of the adlayer material: a high RI analyte can lead to a significant effective index variation and wavelength shift even with a thin adlayer at the surface. Thus, surface sensitivity is usually defined for a specific molecule of interest and is not suitable for a general comparison among sensors operated with different biosensing assays.

The DL is typically specified as the minimum RI (or smallest mass) change necessary to cause a detectable change in the output signal, and defined as follows:

(5) $$DL\;=\;\frac{3\sigma }{S}$$

where σ is the system noise floor, and S is the bulk or surface sensitivity. Since σ depends on the experimental setup and readout instrumentation, this DL is also regarded as the system detection limit (sDL). For an evanescent field label-free biosensor, DL can be specified in three units: (i) DL in units of refractive index units (RIU) aims to characterize the sensing capability in bulk solution, which offers a rough comparison among different sensors; (ii) DL in units of pg/mm^2^ aims to characterize the sensing capability at sensor’s surface by using surface mass density; and (iii) DL in units of ng/mL aims to characterize the sensing capability at sensor’s surface by sample concentrations [15]. Due to the correlation among these DLs, the sensing capability of optical biosensors based on different bioassays can be investigated and compared.

### 2.2. Optical Biosensor Configurations

We select the following representative optical structures that have been reported in the literature and widely used as silicon photonic label-free biosensors at the operating wavelength of visible and near-infrared light.

#### 2.2.1. Interferometer Based Biosensors

**Figure 2:**
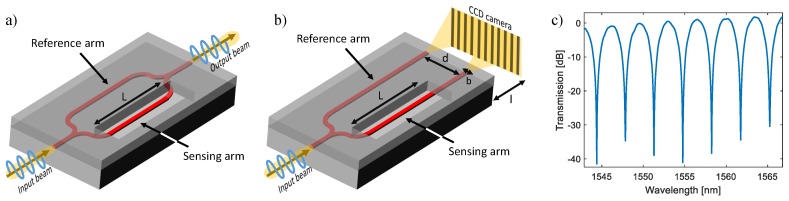
Interferometric biosensors. (**a**) Illustration of a typical Mach–Zehnder interferometer. The light is split into two arms (sensing and reference) and recombined at the output by on-chip Y-junctions. The degree of interference is proportional to the RI variation taking place on the sensing arm. (**b**) Illustration of a classic Young interferometer. Rather than using Y-junctions to rejoin the split beams, the light is projected from two closely spaced secondary sources onto a charge-coupled device (CCD) camera, resulting in an interference pattern. (**c**) Measured interferogram of a typical Mach–Zehnder interferometer (MZI) device after normalization by eliminating the insertion loss.

Interferometer-based biosensors constitute one of the most sensitive integrated-optic approaches by combining two very sensitive methods: waveguiding and interferometry techniques [31]. In a conventional interferometric biosensor, the guided light is split by a Y-junction into two single-mode waveguide paths, one containing the sample, which is regarded as a sensing arm, and the other is used as a reference arm. The evanescent field of the sensing arm interacts with the sample and senses the RI change at the surface, resulting in an optical phase shift. After a certain distance, the beams recombine again and cause a constructive or destructive interference at the output (as shown in Figure 2c), where the intensity modulation corresponds to the RI difference between sample and reference arms.

Young and Mach–Zehnder interferometers are the most common formats for interferometric sensing techniques [27,31,32]. Since the first double-slit experiment by Thomas Young in 1801 [33], and the demonstration of the phase shift detection between two collimated beams by Ludwig Zehnder [34] and Ernst Mach [35] in 1891 and 1892, Young and Mach–Zehnder interferometric configurations have been exploited in biosensors successfully. Although both interferometers utilize Y-junctions to split the coherent, single mode and polarized light at the input, the output recombination of Young interferometers (YIs) is not realized as in MZIs (Figure 2a) by another on-chip Y-junction. Instead, the interference light in YIs is projected on a screen or charge-coupled device (CCD) camera in an off-chip way, as shown in Figure 2b.

In the case of a MZI sensor, the output intensity ($I_{\mathrm{out}}$) is a periodically oscillating function of the phase change difference (Δϕ) of the beams from two arms with the following expression [36]:

(6) $$I_{\mathrm{out}}\;=\;I_{\mathrm{sen}}+I_{\mathrm{ref}}+2\sqrt{I_{\mathrm{sen}}I_{\mathrm{ref}}}\cos\left(\Delta \phi +\Delta \phi_{0}\right)$$

where $I_{\mathrm{sen}}$ and $I_{\mathrm{ref}}$ are the intensity of the light passing through the sensing and reference arms of the MZI, respectively, and $\Delta \phi_{0}$ is the initial phase difference due to the unbalance of the two arms. The phase difference caused by the variation of the effective index ($\Delta n_{\mathrm{eff}}$) at the wavelength λ is calculated as:

(7) $$\Delta \phi \;=\;\frac{2\pi }{\lambda }\Delta n_{\mathrm{eff}}L$$

where *L* is the effective detection length of the sensing arm. As for the YI sensor, since not a single intensity, but an interference pattern (so-called interferogram) is detected at the output, the optical path length difference from two secondary sources is varying along the propagation direction (*y*-axis) [31]. Thus, Equation (6) should be rewritten for YI sensors as [37]:

(8) $$I_{\mathrm{out}}\left(y\right)\;=\;\frac{\sin^{2}\left(b\pi y/\lambda l\right)}{{\left(b\pi y/\lambda l\right)}^{2}}\left[I_{\mathrm{sen}}+I_{\mathrm{ref}}+2\sqrt{I_{\mathrm{sen}}I_{\mathrm{ref}}}\cos\left(\frac{\lambda l}{2\pi d}y+\Delta \phi +\Delta \phi_{0}\right)\right]$$

where *b*, *d* and *l* are the width of a single slit, the distance of two secondary sources and the distance from sources to the detector surface, respectively (as shown in Figure 2b). In this case, the phase difference is expressed as:

(9) $$\Delta \phi \;=\;\frac{2\pi }{\lambda }\left(xd/l-\Delta n_{\mathrm{eff}}L\right)$$

where *x* denotes the position of the interferogram on the camera. The fringe pattern moves laterally at the output. The sensitivity of interferometric sensors is defined as the change in phase caused by the change in the RIU of the cladding above the sensing arm. According to Equations (7) and (9), a longer interaction length (*L*) in the sensing arm can increase the sensitivity [38]. However, due to the cosine-dependent intensity function of the interferometric curve, the intensity response is non-linear: a higher signal change at the quadrature point is observed than the one near the curve extreme of the cosine function. Moreover, false positive signals occur when input source fluctuations or temperature variations happen, which strongly influence the reliability of the interferometric sensor, especially with long sensing arms [39]. Thus, additional modulation approaches are usually needed to tune the phase difference between the arms for interferometer sensors.

The first biosensing application using integrated MZIs was reported by Heideman et al. in the early 1990s [40,41]. Since then, remarkable progress has been achieved in the development of MZI sensors. Different configurations with a variety of fabrication materials including Si_3_N_4_ [41,42], SiO_2_ [43], Si [44,45], polymers [46,47], and even liquid [48] were employed successfully, showing a DL down to 10^−6^ ∼ 10^−7^ RIU. In parallel, chip-integrated YIs have also shown the ability of biomolecule measurement, yielding a comparable DL to the MZI sensor [49,50]. In 2000, a follow-up work by Brandenburg et al. reduced the DL of YI sensors to 9 × 10^−8^ RIU by employing silicon oxynitride as waveguides [51]. Seven years later, Ta_2_O_5_-based YIs have been reported by Schmitt et al. to further improve the sensing ability, with the lowest published DL of 9 × 10^−9^ RIU [52]. Moreover, polymeric materials were also applied to YI sensors in the last few years, which offer a low-cost, mass-produced manufacturing method with a satisfactory sensitivity [53,54].

More recently, Lechuga et al. introduced a BiModal waveguide (BiMW) interferometer for biosensing applications [39,55,56]. Instead of splitting the beam into different arms, the light excites two different modes by a step-junction, and molecular interactions are monitored by the bimodal section. Due to the difference of modal overlap with the analyte, phase changes in two modes introduced by the RI change are distinct, leading to the interference between the two guided modes. The reported DL of the BiMW sensor is as low as 2.5 × 10^−7^ RIU [55], comparable to other interferometric sensors. However, these devices usually need a large footprint, around 5–10 mm in length, which limits the density of on-chip sensors for multiplexable detections.

#### 2.2.2. Resonant Microcavity Based Biosensors

**Figure 3:**
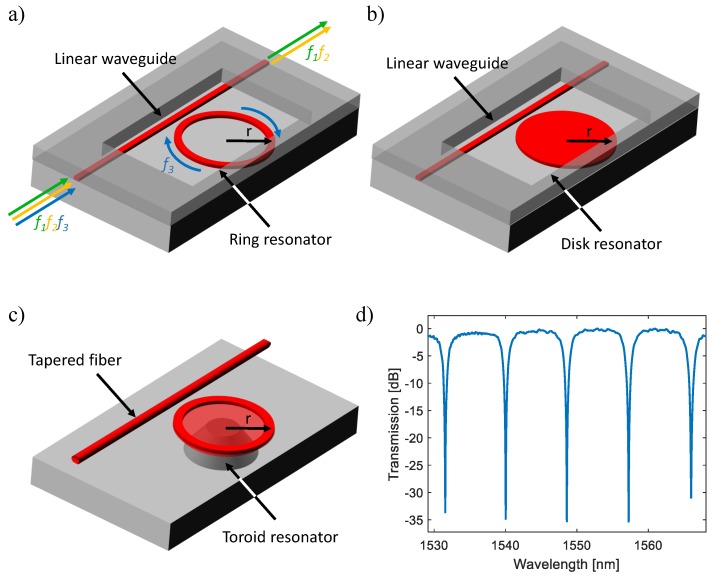
Planar resonant microcavity biosensors. (**a**) Illustration of a conventional microring resonator (MRR) sensor. By using a bus waveguide, guided light is coupled into the resonator at a frequency corresponding to the resonant condition. (**b**) Illustration of a microdisk resonator sensor. (**c**) Illustration of a microtoroid resonator sensor. This structure is coupled by a low-loss tapered fiber, exhibiting an ultrahigh *Q*-factor over 10^8^ [80]. (**d**) Measured transmission spectrum of a conventional MRR device after normalization.

Optical microcavity resonators have been investigated as an emerging sensing technology due to their potential for highly-compact sensing arrays. In a microcavity resonator structure, incident light propagating in an input waveguide or tapered fiber is coupled into the microcavity via the evanescent field. Then, coupled light passes through the cavity in the form of whispering gallery modes (WGMs) or circulating waveguide modes with multiple round-trips, resulting in optical interference at specific wavelengths of light, as shown in Figure 3d by the resonant condition:

(10) $$\lambda \;=\;\frac{2\pi r\times n_{\mathrm{eff}}}{m}$$

where λ is the resonant wavelength, *r* is the radius of the resonator, $n_{\mathrm{eff}}$ is the resonator effective refractive index, and *m* is an integer. The positions of resonant peaks are related to the RI near the resonator surface and shift due to the change of $n_{\mathrm{eff}}$, which can be monitored by scanning the wavelength or by measuring the intensity at a single wavelength.

Unlike interferometric biosensors, the interaction of light and analyte is no longer determined by the length of the sensing waveguide, but rather by the characteristic time of the energy stored inside the resonator, which is characterized by the quality factor (*Q*-factor) [15]. *Q*-factor describes the photon lifetime in the resonator and represents the number of oscillations before the energy has decayed to 37% (1/e). Therefore, *Q*-factor incorporates the distributed loss of a resonator and is approximated by dividing the resonant wavelength by its full width at half maximum (FWHM) [29]:

(11) $$Q\;=\;\omega \frac{\varepsilon }{\partial \varepsilon /\partial t}\;=\;\frac{2\pi n_{g}\times 4.34}{\lambda \times \alpha_{\left(\mathrm{dB}/m\right)}}\approx \frac{\lambda }{\Delta \lambda_{\mathrm{FWHM}}}$$

where ω is the resonant frequency, ε is the energy of the resonant mode, $n_{g}$ is the group index, α is the total distributed loss in the resonator, and $\Delta \lambda_{\mathrm{FWHM}}$ is the FWHM bandwidth of the resonance peak. A higher *Q*-factor indicates that light stays in the resonator longer and interacts more with the analyte. Moreover, White et al. proved that having a high *Q*-factor is advantageous in reducing the noise of the sensor (σ), which further improves the DL [57]. As mentioned before, the DL (or sDL) relies much on the measurement system including curve fitting methods and limitations from light sources or detectors, which makes it difficult to have an objective comparison between sensors with different assays and experimental systems [58]. Consequently, intrinsic detection limit (iDL) was introduced as a substitute for resonant sensors, which is only dependent on intrinsic characteristics, i.e., the resonance linewidth, and defined by Yoshie et al. [59]:

(12) $$iDL\;=\;\frac{\lambda }{Q\times S}$$

where λ, *Q*, and S are the sensor’s resonant wavelength, quality factor, and sensitivity, respectively. By replacing S with S_bulk_ or S_surf_, the bulk or surface iDL can be represented.

Several types of planar resonant microcavity-based configurations have been implemented so far for biosensing since the introduction from two theoretical papers in 2001 [60,61], such as microring (MRR) [62], microdisk [63] and microtoroid [64] shaped resonators (Figure 3). Similar to interferometers, microcavity resonators can be made of Si_3_N_4_ [65,66], SiO_2_ [67,68], Si [18,69], and polymer [70,71] as well. Although resonator-based biosensors enable dense on-chip integration and offer a similar DL of 10^−5^ ∼ 10^−7^ RIU [18,72], their *Q*-factors (except toroid resonators) are relatively low especially with water cladding (around 10^4^) due to the high optical loss, such as side-wall scattering, bend radiation, mode mismatch and material absorption [73]. Microsphere-based ring resonators [74,75] and capillary-based opto-fluidic ring resonators (OFRR) [76] have been recently introduced, supporting improved *Q*-factors over 10^6^ with DLs on the order of 10^−7^ RIU, and applied in a wide sensing range from pesticide [77], cancer [78], to bacteria [79]. However, due to three-dimensional architectures, these devices are not suitable for on-chip fabrication and microfluidics integration. Besides, optical interrogation of these resonators requires meticulous positioning of optical fibers with nanometer precision and alignment [27].

#### 2.2.3. Photonic Crystal Based Biosensors

Porous silicon (PSi) has been applied as the optical sensor for the detection of chemicals and molecular interactions since 1997 [81]. By using electrochemical etching of crystalline silicon in HF-based solutions, as well as physical, physicochemical, chemical and electrochemical post-procedures, various PSi layers have been developed and established [82]. Thanks to the porous nature of PSi architectures, an extremely high surface area within a small volume is achieved with narrow optical reflectivity features, which offers a decreased DL with enhanced sensitivities compared to Fabry–Perot based optical sensors [83]. Photonic crystals (PhC) and Bragg reflectors are two main configurations developed by PSi for biosensing purpose. In this review, we focus on the next generation of PSi sensors, waveguide-based PhC and Bragg devices appeared around 2009 [84,85], which provide more optical confinement and guidance within their planar waveguides.

**Figure 4:**
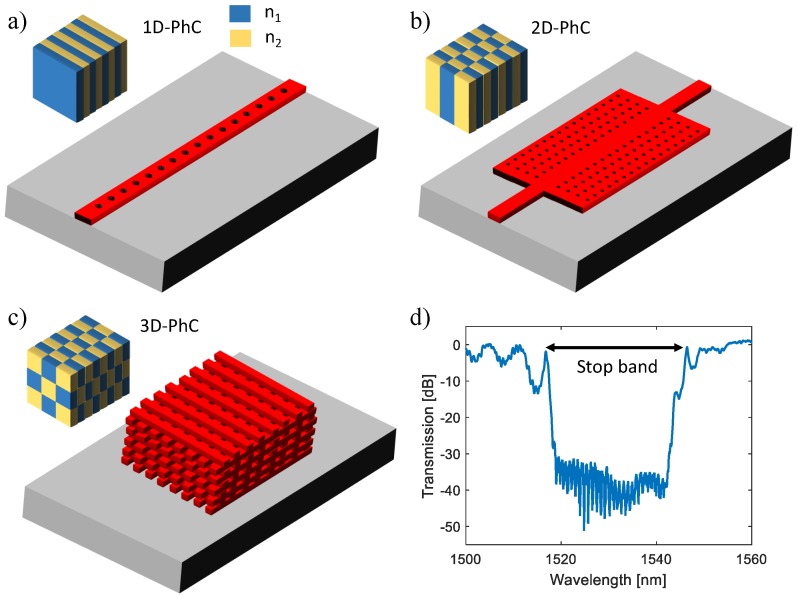
Illustration of photonic crystals in: (**a**) 1D conformation; (**b**) 2D conformation; and (**c**) 3D conformation. Insert: Schematic representation of each format showing the periodic arrangements, different colors represent materials with different indices. (**d**) Measured transmission spectrum of a uniform photonic crystal (PhC) device after normalization.

A photonic crystal (PhC) waveguide consists of periodically repeating arrays of dielectric structures, forming periodic variations in the refractive index. The periodicity is on the order of the optical wavelength and stops a range of wavelengths propagating through the PhC, resulting in a photonic bandgap on the transmission (or reflection) spectrum presented in Figure 4d. By introducing a defect into the PhC structure, a defect mode at a particular wavelength is formed and resonantly confined in the defect region, which leads to a sharp peak within the bandgap. Due to the strong optical confinement, light is concentrated in a minimal volume near the defect, enabling an intense light–matter interaction area. A tiny volume of analytes immobilized surrounding the defect can induce a noticeable shift of the resonance wavelength and provide a measurable response. Hence, in the past ten years, PhC based biosensors are regarded as a promising and novel technology that has gained much attention [86,87,88].

The periodicity of a PhC structure can be one-dimensional (1D), two-dimensional (2D) or three-dimensional (3D). One-dimensional PhCs are the most straightforward architecture analyzed by Lord Rayleigh as early as 1887. These structures consist of different material layers with high and low refractive indices alternatively (Figure 4a) and are usually fabricated by layer-by-layer deposition, spin coating, or photolithography methods [89]. In 1987, Yablonovitch [90] and John [91] reported the detailed research on PhCs separately, proposing the concept of photonic bandgaps in 2D and 3D structures. 2D and 3D PhCs exhibit their periodicity in two and three spatial directions as shown in Figure 4b,c, which need complex manufacturing techniques such as photolithography, etching, particle self-assembly, etc [89]. Although the complexity of the manufacturing process of 1D PhC devices is low, a well-collimated beam is usually required for sensing approaches, especially for high *Q*-factor devices, which needs the sensing area to be relatively large, compared to 2D or 3D ones [92].

PhC biosensors were first developed using TiO_2_-coated polymer gratings by Cunningham et al. in the early 2000s, offering an inexpensive manufacturing technique on plastic films [93,94,95]. At the same time, Si-based PhC devices in the SOI platform were also investigated and have developed rapidly leveraging electron beam lithography (e-beam) technology, including 1D PhC [96,97,98], 2D PhC [25,86] based architectures, for biomolecule detections. Chow et al. demonstrated an ultra-compact PhC sensor with a sensing area of 10 µm^2^, enabling a DL of better than 2 × 10^−3^ RIU and a *Q*-factor of 400 in 2004 [86]. Later, in 2010, Skivesen et al. achieved an improved DL of 6.75 × 10^−4^ RIU by tracking sharp fringes appearing in the slow-light regime near the edge of the guided band [99]. In the same year, Kang et al. increased the sensing surface area to the defect region of PhCs by introducing multiplehole defects (MHDs), showing an enhanced sensitivity compared to PhCs with single hole defects (SHDs) [100,101]. Qin et al. incorporated the concept of MHDs to the slow-light MZI-based biosensor, showing a thirteen-fold higher bulk sensitivity than traditional MZI biosensors of 115,000 rad/RIU-cm [102]. Lo et al. announced an optical biosensor based on a 1D-PhC microring resonator (PhCR) with enhanced detection sensitivity in 2017 [103]. By introducing the 1D PhC geometry in a MRR’s waveguide, the light–matter interaction is strongly improved since the PhCR can detect the presence of analyte both inside 1D holes and on the top surface [103].

Compared to interferometric or other resonant biosensors, PhC sensors tend to have lower sensitivities ranging from 10^−2^ to 10^−4^ RIU. However, PhC sensors can be readily integrated onto a chip with high density, and are suitable for detection with extremely limited sample volumes (on the order of femtoliter). Therefore, a new trend of PhC sensor development is to achieve multi-analyte detection capability on a single chip. Several 1D and 2D PhC-based sensor arrays were developed [85,104,105,106]. In 2017, Zhang et al. designed a highly sensitive on-chip multichannel sensor array by integrating eight 1D PhC cavities connected by additional bandgap filters, showing improvements in size, integration density, sensitivity, and ease of fabrication [107].

#### 2.2.4. Bragg Grating Based Biosensors

**Figure 5:**
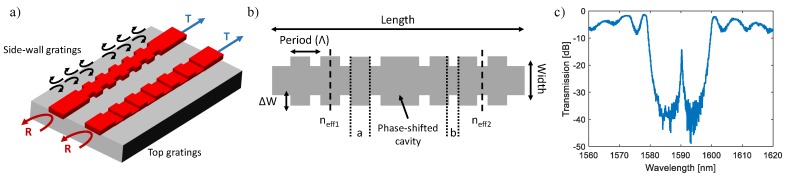
Bragg grating biosensors. (**a**) Illustration of two types of Bragg grating devices with side-wall or top gratings. R and T are the grating’s reflection and transmission. The 180° arrows represent the numerous reflections throughout the grating. (**b**) Schematic of a phase-shifted Bragg grating device. Λ is the period, ΔW is the width of the corrugation, a or b and $n_{\mathrm{eff}1}$ or $n_{\mathrm{eff}2}$ are the length and the effective index of the high or low index section. (**c**) Measured transmission spectrum of a phase-shifted Bragg grating device after normalization.

The Bragg grating, a fundamental component for the purpose of wavelength selection, has been investigated for use in optical communications, such as filters, semiconductor lasers and fibers for a long time [73], and recently into biosensing applications [22,108]. Similar to 1D photonic crystals, a Bragg grating is a structure with a periodic modulation of the effective RI in the propagation direction of the optical mode, as shown in Figure 5. By alternating the material with different indices or physical dimensions (known as the corrugation) of the waveguide, the desired index modulation is achieved. A reflection of the guided light occurs at each index-changed boundary as presented in Figure 5a, and the repeated modulations of the effective index multiply the distributed reflection, resulting in a stop band at one specific wavelength in the transmission spectrum, where light is strongly reflected. The center wavelength of the stop band, namely the Bragg wavelength, is given as:

(13) $$\lambda \;=\;2\Lambda \times n_{\mathrm{eff}}$$

where Λ is the period, and $n_{\mathrm{eff}}$ is the average effective index of Bragg gratings. If a phase-shifted cavity is introduced in the middle of the gratings, as illustrated in Figure 5b, a narrow resonant transmission peak will appear within the stop band [109], which can be utilized for RI change monitoring.

Fiber Bragg gratings (FBGs) have attracted a great deal of attention in recent years for biosensing applications, due to the low price and ease of signal transmission of fiber materials. To improve the sensing performance, numerous studies have been attempted to expose the evanescent field from the fiber core, such as side-polishing or surface-etching strategies, achieving a DL down to 10^−5^ ∼ 10^−6^ RIU [110,111,112]. Recent advances in Bragg gratings have led to the on-chip integration realized in the SOI platform, firstly demonstrated by Murphy et al. in 2001 [113]. A theoretical demonstration of biosensing capability of SOI-based Bragg gratings was announced by Passaro et al. in 2008 [114]. By periodically etching the top surface of the silicon waveguide, a submicrometer integrated optical Bragg grating sensor is proposed with a simulated DL of approximately 10^−4^ RIU [114]. One year later, Jugessur et al. developed a uniform Bragg grating biosensor integrated with microfluidics for RI index sensing by using vertical grating side-edges proving potential for lab-on-a-chip applications [22]. Prabhathan et al. proposed the concept of a phase-shifted vertical side wall gratings for biosensing in the same year with a theoretical DL of 8.1 × 10^−5^ RIU [23]. In 2013, Fard et al. fabricated and characterized the strip-waveguide based phase-shifted Bragg grating in the SOI platform, and the *Q*-factor was measured to be 27,600, which led to a experimental iDL of 9.3 × 10^−4^ RIU [115].

### 2.3. Section Summary

**Figure 6:**
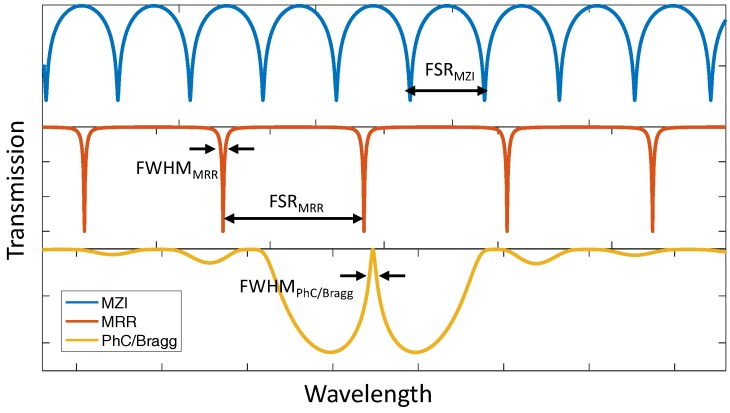
Simulated transmission spectra of different optical configurations, including MZI (blue curve), microring resonator (MRR) (red curve), defected PhC or phase-shifted Bragg grating (yellow curve) sensors. The optical insertion loss caused by input and output coupling devices has been eliminated. The full width at half maximum (FWHM) indicates the optical wavelength width of the resonant peak at which the transmitted intensity is equal to half (−3 dB) of its maximum value.

Figure 6 summarizes the simulated transmission spectra of previously described optical configurations in the field of silicon photonic biosensors. As a concept illustration, we only consider the intrinsic losses in each device. As shown in Figure 6, MZI (blue curve) and MRR (red curve) sensors present periodic spectra. The spacing between optical wavelengths of two consecutive transmitted optical intensity minima is defined as the free spectral range (FSR) and given by:

(14) $$\Delta \lambda_{\mathrm{FSR}}\;=\;\frac{\lambda^{2}}{n_{g}\times \Delta L}$$

where λ is the wavelength of the light source, $n_{g}$ is the waveguide group index, and ΔL is the length difference of two arms in the MZI or the perimeter of the MRR. As for the transmission spectrum of the PhC or Bragg grating (yellow curve), due to the existence of the defect or phase-shifted cavity, a sharp FSR-free resonant peak appears in the middle of the stop band with a narrow FWHM corresponding to the high *Q*-factor. By interrogating the wavelength (phase) shift or intensity change of these peaks in the transmission plots, the RI change caused by the analyte within the evanescent field can be monitored in real-time.

Generally, compared to other geometries, the MZI-based optical sensor is one of the simplest configurable devices with better sensitivities that scale with the length of the sensing arm. As described in Equations (1) and (3), the sensor structure constant *K* in a feedback-based (such as MRRs) sensor is 1, whereas, in an feedforward-based (such as MZIs) sensor, *K* equals to $L_{1}/\left(L_{1}-L_{2}\right)$ where $L_{1}$ and $L_{2}$ are waveguide lengths of sensing and reference arms, respectively. That can be derived in a physical way by introducing a perturbation parameter *q* into the sensing system, as described in Ref. [116], which leads to the propagation constant change of the waveguide ($\Delta \beta_{q}$), thus changes the wavelength of the resonant condition or destructive interference in MRR or MZI sensors. Further changes in the propagation constant happen due to the wavelength shift ($\Delta \beta_{\lambda }$). In the MRR-based sensor, phase changes of one round-trip in the cavity before ($\phi_{1}$) and after ($\phi_{2}$) the perturbation *q* follow:

(15) $$\phi_{1}\;=\;\beta L\;=\;\phi_{2}\;=\;\left(\beta +\Delta \beta_{q}+\Delta \beta_{\lambda }\right)L\;=\;2m\pi$$

where *L* is the resonant cavity length, β is the initial propagation constant, and *m* is an integer. After the derivation of λ, we get:

(16) $$\Delta \lambda_{\mathrm{MRR}}\;=\;\frac{\lambda \times \Delta n_{\mathrm{eff}}}{n_{g}}.$$

In the case of MZI-based sensors, phase changes due to the destructive interference between two arms are described (only the sensing arm is influenced by *q*):

(17) $$\phi_{1}\;=\;\beta_{1}L_{1}-\beta_{2}L_{2}\;=\;\phi_{2}\;=\;\left(\beta_{1}+\Delta \beta_{1,q}+\Delta \beta_{1,\lambda }\right)L_{1}-\left(\beta_{2}+\Delta \beta_{2,\lambda }\right)L_{2}\;=\;\left(2m-1\right)\pi$$

where $\Delta \beta_{1,q}$ is the propagation constant change caused by *q* in the sensing arm, $\beta_{1}$ and $\beta_{2}$ are initial propagation constants, $L_{1}$ and $L_{2}$ are waveguide lengths, and $\Delta \beta_{1,\lambda }$ and $\Delta \beta_{2,\lambda }$ are propagation constants change due to λ of sensing and reference arms, respectively. It can be shown that:

(18) $$\Delta \lambda_{\mathrm{MZI}}\;=\;\frac{\lambda \times \Delta n_{\mathrm{eff}}L_{1}}{n_{g1}L_{1}-n_{g2}L_{2}}\approx \left(\frac{L_{1}}{L_{1}-L_{2}}\right)\frac{\lambda \times \Delta n_{\mathrm{eff}}}{n_{g}}.$$

Therefore, the sensitivity is independent of the physical size in a MRR-based sensor, but scales with the length ratio between the sensing arm and arms difference in a MZI-based counterpart, as presented below:

(19) $$S_{\mathrm{MRR}}\;=\;\frac{\Delta \lambda_{\mathrm{MRR}}}{\Delta n_{\mathrm{add}}}\;=\;\frac{\lambda }{n_{g}}\left(\frac{\partial n_{\mathrm{eff}}}{\partial n_{\mathrm{add}}}\right),$$

and

(20) $$S_{\mathrm{MZI}}\;=\;\frac{\Delta \lambda_{\mathrm{MZI}}}{\Delta n_{\mathrm{add}}}\;=\;\left(\frac{L_{1}}{L_{1}-L_{2}}\right)\frac{\lambda }{n_{g}}\left(\frac{\partial n_{\mathrm{eff}}}{\partial n_{\mathrm{add}}}\right)\;=\;\frac{2\pi L_{1}}{\lambda }\left(\frac{\partial n_{\mathrm{eff}}}{\partial n_{\mathrm{add}}}\right)\;=\;\frac{\Delta \phi_{\mathrm{MZI}}}{\Delta n_{\mathrm{add}}}.$$

In terms of the detection limit, the concept of intrinsic DL has been mentioned for MRR-based sensors in Equation (12), which only depends on the silicon photonic device itself. In Reference [14], the FWHM of the resonance spectrum for an all-pass MRR is:

(21) $$\Delta \lambda_{\mathrm{FWHM}}\;=\;\frac{\left(1-ra\right)\lambda^{2}}{\sqrt{ra}\times n_{g}\pi L}$$

where *a* is the single-pass amplitude transmission ($a^{2}\;=\;\exp\left(-\alpha \times L\right)$, and α is the power attenuation [1/cm]), and *r* is the self-coupling coefficient. The iDL of a MRR-based sensor is achieved by combining Equations (11), (12) and (19):

(22) $$iDL_{MRR}\;=\;\frac{\Delta \lambda_{\mathrm{FWHM}}}{S}\;=\;\frac{(1-ra)\lambda }{\sqrt{ra}\times \pi L}\left(\frac{\partial n_{add}}{\partial n_{eff}}\right).$$

However, for MZI-based sensors, no such metrics are proposed. That is due to the sinusoidal shape of the interferometric spectrum, which fixes the linewidth of the FWHM to be half of the FSR and is independent of the loss. Hence, in a MZI-based sensor, if we derive in the same way, iDL is only related to its sensitivity, i.e., to the length of its sensing arm ($L_{1}$):

(23) $$iDL_{\mathrm{MZI}}\;=\;\frac{\lambda }{2L_{1}}\left(\frac{\partial n_{add}}{\partial n_{eff}}\right).$$

Disadvantages such as large footprint, high-temperature sensitivity, and the need for additional modulation methods hinder the development of on-chip interferometric sensing arrays. Resonator-based sensors, such as MRRs, microdisks, PhCs and Bragg gratings, are more suitable for the integrated sensing platform with a high density due to their small sizes. Different from MRRs, PhCs and Bragg gratings have a high *Q*-factor due to the elimination of bending (mode and radiation) losses, thus an improved iDL, even though their sensitivities are comparable. Although silicon-based architectures have been successfully applied for the detection of cell secretions [117], virus [118], protein biomarkers [11], and nucleic acids successfully [119,120], a lower detection limit with a higher sensitivity is still required for current clinical diagnostic tests [121].

## 3. Performance-Improving Strategies

In this section, we outline early and emerging strategies in the development of SOI-based biosensor performance, including the use of new geometries of optical waveguides, and different polarizations or wavelengths of light sources. Furthermore, an overall performance metrics comparison is presented at the end, which includes proposed sensing architectures with or without their performance improved strategies.

### 3.1. Fundamental Approaches

#### 3.1.1. Transverse Magnetic Mode

**Figure 7:**
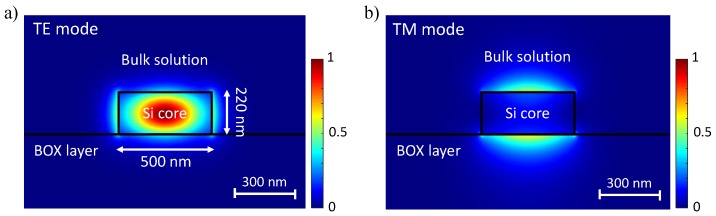
Illustration of electric field intensity distributions of the (**a**) quasi-transverse electric (TE) and (**b**) quasi-transverse magnetic (TM) modes in a 200 × 500 nm silicon waveguide at 1550 nm wavelengths. The Si waveguide core ($n_{\mathrm{eff}}$ = 3.47) is exposed to the surrounding medium with a refractive index of 1.33 above a 2 µm thick buried oxide layer (BOX) with a refractive index of 1.44.

Due to the large evanescent field component traveling above the waveguide, optical sensors in the quasi-transverse magnetic (TM) mode present an improved sensitivity to that of the quasi-transverse electric (TE) mode at 1.55 µm in conventional 220 nm-thick SOI waveguides [44,122]. Figure 7 below shows the electric field intensity distributions of the TE and TM modes propagating in a 220 × 500 nm waveguide. Most of the field intensity is above and beneath the waveguide core (in the cladding and substrate) in the TM mode, offering a higher light–matter interaction strength. Moreover, the TM mode also experiences less scattering loss, which is usually caused by sidewall roughness, compared to the TE mode [30]. Because of these unique properties of TM mode based waveguides, a large number of evanescent field biosensors have been attempted in the TM mode for higher susceptibility to RI changes.

For the MZI configuration, Densmore et al. made many contributions to surface biosensing by introducing TM polarized light [17,45,123]. These TM mode based MZI biosensors achieved a minimum detectable mass of ∼10 fg of streptavidin [17] and ∼0.5 fg of anti-rabbit IgG [45], respectively. In 2008, Zinoviev et al. developed a MZI-based biosensor by using Si_3_N_4_, where the lowest DL in the variation of the RI for the TM polarization is found to be 10^−7^ RIU [12]. Similarly, TM mode based resonant microcavities have been investigated as alternatives to their TE mode counterparts. An investigation of silicon MRR based biosensor arrays was reported by Xu et al. in 2010 with an experimental sensitivity of 135 nm/RIU; binding interactions between complementary IgG protein pairs was monitored with a concentration down to 20 pM by utilizing TM-polarized light [124]. Fard et al. reported a sensitivity enhanced TM mode MRR biosensor by decreasing the thickness of silicon waveguides to 150 nm, resulting in sensitivities as high as 270 nm/RIU and 437.5 pm/nm for bulk and surface analytes [19]. In 2013, Grist et al. introduced Si-based microdisk resonators for label-free biosensing, and experimental results showed sensitivities of 26 nm/RIU and 142 nm/RIU, and *Q*-factors of 3.3 × 10^4^ and 1.6 × 10^4^ for the TE and TM modes, respectively [21].

#### 3.1.2. Slot Waveguides

**Figure 8:**
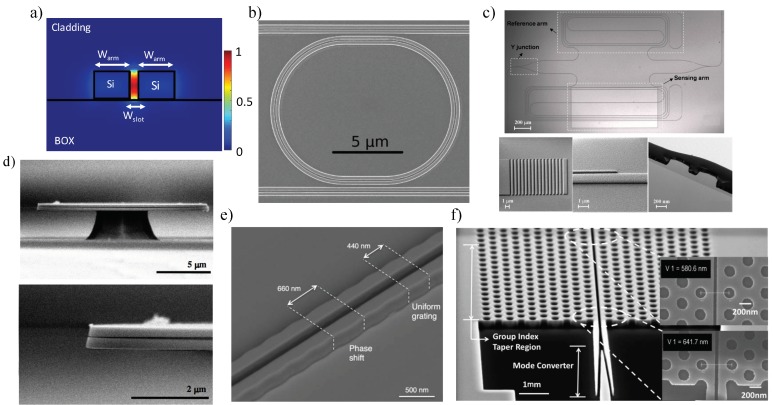
(**a**) Cross-section of the electric field intensity distribution of a slot-waveguide immersed in water. (**b**) Top-view scanning electron microscope (SEM) image of the slot-waveguide-based MRR. Figure adapted with permission from Reference [128]. (**c**) Microscopic and SEM images of the MZI biosensor with a slot-waveguide sensing arm. Figure adapted with permission from Reference [134]. (**d**) SEM images of the fabricated slot disk after the whole sensing process. Figure adapted with permission from Reference [132]. (**e**) SEM image showing a phase-shifted Bragg grating sensor, the spacing with the phase shift is 600 nm, corresponding to 1.5 times the grating period. Figure adapted with permission from Reference [135]. (**f**) SEM images of fabricated PC slot-waveguide device, showing a slot entirely across the device. Figure adapted with permission from Reference [136].

A slot-waveguide device consists of two high index rails separated by a low index slot [65]. Because of the high concentration of the electric field intensity within the slot, slot-waveguide based structures stand out for the potential to enhance sensitivity for optical biosensors. As presented in Figure 8a, light is strongly confined in the slot region. Thus, compared to conventional waveguides, a stronger light–matter interaction can be obtained in this region, leading to an improved sensitivity. In addition, slot-waveguide based structures are also CMOS compatible which enables miniaturization and integration for a lab-on-a-chip platform with low cost [38,125].

In 2005, Baehr-Jones et al. designed, fabricated and characterized MRRs based on slot-waveguide geometries in SOI materials [126]. Two years later, Barrios et al. pioneered the development of slot-waveguide biosensors by using Si_3_N_4_-based MRRs with a slot width of 200 nm for both the waveguide and resonator [127]. A highly improved bulk sensitivity of 212 nm/RIU with a *Q*-factor of 1800 and DL of 2 × 10^−4^ RIU is achieved [127]. In 2010, an integrated optical Si_3_N_4_ slot-waveguide MRR sensor array was reported by Carlborg et al. for multiplexed label-free biosensing, yielding a bulk DL of 5 × 10^−6^ RIU and a surface mass density DL of 0.9 pg/mm^2^ [65]. In the same year, Claes et al. presented a double-bus MRR comprised of SOI-based slot-waveguides with 104 nm slot width (Figure 8b), and obtained a sensitivity of 298 nm/RIU and DL of 4.2 × 10^−5^ RIU for changes in the RI of the top cladding [128]. In 2016, Taniguchi et al. developed MRR biosensors with silicon nitride slot waveguides due to the lower temperature coefficient, achieving a detection of prostate specific antigen (PSA) with the DL of 1 × 10^−8^ g/mL, which is the concentration strongly suspicious for prostate cancer [129]. In the same year, Zhang et al. investigated a racetrack all-pass slot-waveguide MRR showing a V-shaped resonant spectrum modulated by the classical frequency comb, by tracking the spectrum envelope wavelength shift, and an ultra-high sensitivity up to 1300 nm/RIU is received [130]. However, the sensing strategy is based on the wavelength-sensing critical coupling condition, which makes the sensitivity very wavelength dependent. A horizontal slot waveguide configuration was proposed by Barrios for Si-based microdisk resonator biosensors for the TM polarization in 2006, showing an expected *Q*-factor of 15,000 with a minimum DL of 3 × 10^−8^ RIU [131]. Four years later, Lee et al. followed up that concept and demonstrated a horizontal air-slot microdisk resonator for label-free biosensing based on silicon nitride as shown in Figure 8d, obtaining a *Q*-factor of 7000 in the TM mode with a DL of 30 ng/mL for biotin–streptavidin interactions [132]. Kim et al. reported a luminescent horizontal air-slot microdisk resonator sensor based on silicon-rich nitride (SRN) in the 800-nm wavelength range, achieving a surface sensitivity of 4.79 nm/(µm-mL) by introducing biotin–streptavidin model [133].

Slotted PhCs combine the advantages of light confinement in the slot waveguide with the temporal confinement of light by a PhC in a single structure, offering more light interactions with the analyte [137]. Di Falco et al. reported a sensitivity improved (over 1500 nm/RIU) label-free biosensor by applying a PhC to slot geometry with a high *Q*-factor of 50,000 and DL of 7.8 × 10^−6^ RIU in 2009 [138]. Jágerská et al. and Lai et al. (Figure 8f) expanded the application of slotted PhCs for gas detections, obtaining a DL of 10^−5^ RIU for a variety of gases [139] and a methane concentration of 100 ppm [136], respectively.

Plenty of work has been reported by using MZI devices with slotted sensing arms for the pursuit of a high sensitivity. In 2012, Tu et al. presented an athermal MZI biosensor based on Si_3_N_4_ slot waveguides (see Figure 8c), with a the measured bulk sensitivity and DL reach of 1730(2π)/RIU and 1.29 × 10^−5^ RIU, respectively [134]. One year later, they followed up the investigation for biosensing by using a biotin–streptavidin binding model system, and demonstrated a DL down to 1 pg/mL of streptavidin solutions [140]. Furthermore, they also investigated the biosensor for specific detection by employing the methylation of death-associated protein kinase (DAPK) gene, showing a discriminated concentration as low as 1 nM [140]. In 2015, Sun et al. developed a MZI sensor employing an ultra-compact double-slot hybrid plasmonic (DSHP) waveguide as an active sensing arm [141]. By introducing a DSHP waveguide with two open nano-slots between a high-index Si ridge and two silver strips, a high optical confinement with low propagation loss was achieved, showing a sensitivity as high as 1061 nm/RIU [141].

Recently, Wang et al. presented a slot-waveguide based biosensor using phase-shifted Bragg gratings [135]. As presented in Figure 8e, the Bragg gratings with sidewall corrugations created a sharp resonant peak within the stop band by introducing a phase shift. A salt solutions assay demonstrated a sensitivity of 340 nm/RIU and *Q*-factor of 1.5 × 10^4^, enabling a low iDL of 3 × 10^−4^ RIU [135].

#### 3.1.3. Thinner Waveguides

**Figure 9:**
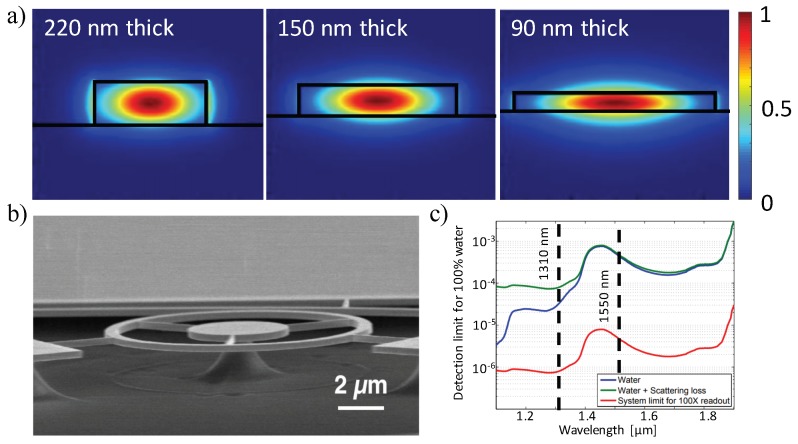
(**a**) Electric field intensity distributions of a TE mode for 90, 150 and 220 nm thick silicon cores. Figure adapted with permission from Reference [30]. (**b**) Tilted SEM image of an MRR after suspension. The MRR is supported by trusses with a width of 100 nm and a height of 260 nm. Figure adapted with permission from Reference [145]. (**c**) Fundamental DL plots for water-based sensors at 1310 and 1550 nm wavelengths. Highest predicted DL for water absorption limited sensing is presented (blue line). Waveguide scattering is added and assumed to contribute 5 dB/cm loss at 1550 nm, and scale as 1/$\lambda^{4}$ at other wavelengths (green line). Finally, the sDL is shown (red line) with a wavelength readout precision 100-fold better than the resonator linewidth. Figure adapted with permission from Reference [29].

Using thinner waveguides can lead to a lower optical confinement of the guided mode, resulting in a deeper penetration of the evanescent field into the surrounding medium, as shown in Figure 9a. Thus, more field overlap with biomolecules at the waveguide’s surface is achieved. In 2006, Densmore et al. theoretically demonstrated that thinner SOI waveguides have higher sensitivities over devices both to bulk homogeneous solutions and thin adsorbed biomolecule layers [44]. Afterward, Fard et al. investigated an ultra-thin TE MRR sensor using the smallest available thickness (90 nm) offered by multi-project wafer (MPW) foundries, obtaining a sensitivity over 100 nm/RIU with the iDL on the order of 5 × 10^−4^ RIU [142]. Moreover, due to the index of the water cladding decreasing with rising temperature which is opposite to the Si core and SiO_2_ substrate materials, ultra-thin TE MRR sensors show increased stability in the presence of temperature variations as compared to the traditional 220 nm thick sensors [142].

#### 3.1.4. Suspended Waveguides

Another method to enhance the overlap between the evanescent field and analyte is introducing suspended waveguides, by replacing the BOX substrate with lower-index materials (e.g., air and water). In 2000, Veldhuis et al. theoretically proposed that the sensing performance can be improved by using a suspended silicon waveguide technology, where the sensitivity is enhanced by a factor of 1.35 [143]. After that, many suspended sensors were reported successively leveraging the SOI platform. Wang et al. demonstrated an ultra-small suspended microdisk with a radius of 0.8 µm sitting on a SiO_2_ pedestal for optical sensing, presenting a measured sensitivity of 130 nm/RIU in 2013 [144]. Soon after, a suspended TM-MRR biosensor to increase the surface binding area and light–matter interaction was reported by Hu et al. (Figure 9b), showing a near three-fold increased response to bulk RI changes (290 nm/RIU) and two-fold increased response to the capture of targets at the surface as compared to conventional MRRs on SiO_2_ (102 nm/RIU) [145]. Taha et al. recently developed a centimeter-scale MZI sensor based on SOI platform by introducing a fully suspended waveguide as the sensing arm, obtaining a bulk sensitivity of 740 nm/RIU with a corresponding iDL of ∼4 × 10^−5^ RIU [146].

#### 3.1.5. 1310 nm Light Sources

For label-free biosensing, one way to improve the limits of detection of silicon photonic sensors for medical diagnostic applications is enhancing the intrinsic sensor performance [30]. According to Equation (12), iDL shows a reciprocal relation to its *Q*-factor and S. Thus, having a large *Q*-factor or sensitivity value can effectively improve the iDL. The *Q*-factor can be interpreted as the total distributed loss of the device based on Equation (11), the loss originates from waveguide scattering, material absorption (waveguide and analyte), waveguide radiation, mode mismatch, etc. [29]. Among them, water absorption is the predominant loss for silicon photonic biosensors at 1550 nm wavelengths since many analytes of interest are found in aqueous solutions. Kou et al. observed that water absorption is approximately 10 times lower around 1310 nm wavelengths compared to 1550 nm ones [147]. By assuming an ideal Fabry–Perot cavity with the light traveling entirely in the water, where no other loss mechanism exists, a fundamental limit for water-based sensors was calculated by Chrostowski et al., showing a intrinsic limit of detection of 2.4 × 10^−4^ RIU at 1550 nm and 2.4 × 10^−5^ RIU at 1310 nm, respectively in Figure 9c [29].

Various silicon photonic biosensors for 1310 nm wavelengths were reported by Schmidt et al. in 2014, including MRRs in the TE and TM modes, and Bragg gratings in the TM mode [30]. Experimental characterizations result in a measured *Q*-factor of 8389, bulk sensitivity of 90 nm/RIU, and iDL of 1.49 × 10^−3^ RIU for the TE mode MRR, and a *Q*-factor of 33,463, bulk sensitivity of 113 nm/RIU, and iDL of 3.47 × 10^−4^ RIU for the TM mode MRR. For TM mode Bragg gratings, a high *Q*-factor of 76,320 with a bulk sensitivity of 106 nm/RIU and iDL of 1.62 × 10^−4^ RIU is achieved. In 2016, Melnik et al. investigated a MZI biosensor based on polyimide waveguides at the central wavelength of 1310 nm for human immunoglobulin G (hIgG) detection, allowing detecting concentrations down to 3.1 nM and 100 pM by label-free and labeled methods, respectively [148].

### 3.2. Advanced Approaches

#### 3.2.1. Sub Wavelength Grating Waveguides

**Figure 10:**
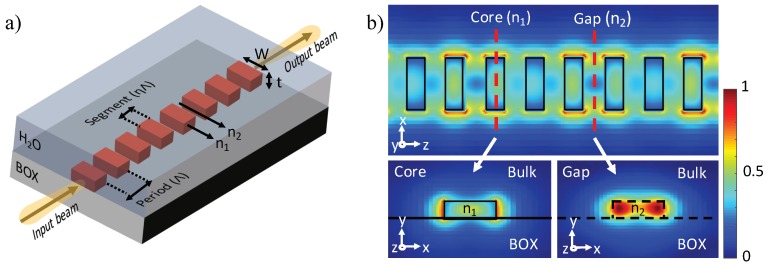
Sub-wavelength grating (SWG) waveguide geometry and simulation results. (**a**) Schematic of an SWG waveguide. *W* is the waveguide width, *t* is the thickness, Λ is the SWG period, and η is the duty cycle which determines the length of Si blocks. $n_{1}$ and $n_{2}$ represent high and low refractive indices. (**b**) The top and cross-sectional views of the electric field intensity distribution of an SWG waveguide. The cross-sections are in the middle of the Si block and gap, respectively.

A novel and appealing strategy that allows customizing optical properties by varying the waveguide geometry is using sub-wavelength gratings (SWG) [149]. Since the first demonstrations of an optical waveguide with an SWG metamaterial core by the National Research Council of Canada (NRC) in 2006 [150,151,152], SWG waveguides have attracted intense research interest due to their unique potentials to control light propagation in planar waveguides, and been considered to be critical components for developing the next generation of optical communication, biomedical, quantum and sensing technologies [153,154]. Although similar to Bragg gratings, SWG waveguides also consist of the periodic structure of their core, the period (Λ) is much smaller than the Bragg condition, i.e., $\Lambda \ll \lambda /(2n_{\mathrm{eff}})$. Thus, a true lossless mode is supported in SWG waveguides because the reflection and diffraction effects are suppressed [155]. The SWG waveguide core is commonly fabricated by interleaving the high index block ($n_{1}$) with low index materials ($n_{2}$), such as SiO_2_, SU-8, air or water, as one period (a few hundred nanometers in length), as shown in Figure 10a. By having a reduced mode effective index step, the guided light propagates in SWG waveguides similar to the one in conventional waveguides but with a large extended modal area, which releases more optical mode into the evanescent field. Moreover, as shown in Figure 10b, most of the light is concentrated in the low-index region which offers direct light–matter contact. Thus, compared to the conventional waveguide, the sensing performance of an SWG waveguide-based biosensor is highly enhanced.

**Figure 11:**
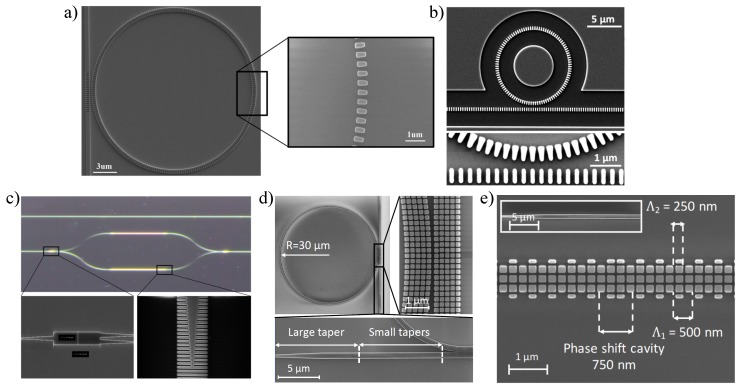
(**a**) SEM images of a fabricated SWG MRR with waveguide geometry: *W* = 500 nm, Λ = 250 nm, *t* = 220 nm, and η = 0.7. Figure adapted with permission from Reference [161]. (**b**) SEM images of a 5 µm radius trapezoidal silicon pillars based SWG MRR, and a high magnification of the coupling region. Figure adapted with permission from Reference [157]. (**c**) Microscopic and SEM images of the fabricated MZI device with an SWG waveguide based sensing arm. Figure adapted with permission from Reference [164]. (**d**) SEM images of a multi-box MRR (*r* = 30 µm, *W* = 1200 nm, *t* = 220 nm, Λ = 240 nm and η = 75%) with five rows. Figure adapted with permission from Reference [162]. (**e**) SEM images of a three-row multi-box phase-shifted Bragg grating sensor with 500 nm Bragg period ($\Lambda_{1}$), 250 nm SWG period ($\Lambda_{2}$), and 120 nm wide corrugations. Figure adapted with permission from Reference [163].

In 2014, Wangüemert-Pérez et al. proposed the application of SWG waveguides for biosensing and employed a Fourier-type 2D vectorial simulation tool to analyze the sensing performance by varying the duty cycle, achieving sensitivities of 0.83 RIU/RIU (the change in the $n_{\mathrm{eff}}$ of the waveguide mode upon a change in the RI of the cover) and 1.5 × 10^−3^ RIU/nm (or for an increase in the thickness of the adsorbed layer) for bulk and surface sensing [156]. After that, Chen’s [157,158,159] and Chrostowski’s [160,161,162,163] groups pioneered the development of SWG waveguide-based biosensors in the SOI platform. Donzella et al. demonstrated SOI-based SWG optical MRRs for integrated optics and sensing in 2015, showing the first time that SWG-based resonators with no upper cladding can achieve sensitivities exceeding 383 nm/RIU in water and 270 nm/RIU in air [160]. A follow-up work was reported by Flueckiger et al. (Figure 11a) by introducing NaCl dilutions and a typical protein bioassay to the SWG MRR sensor, achieving a bulk sensitivity of 490 nm/RIU with a system DL of 2 × 10^−6^ RIU [161]. However, one serious drawback of SWG-based MRR sensors is the relatively low *Q*-factor with the upper cladding removed, which is in the range of 1000∼6000 [160]. Trapezoidal silicon pillars, as reported by Wang et al., can reduce the bend loss by creating an asymmetric effective refractive index profile in the microring (as shown in Figure 11b), yielding a *Q*-factor as high as 11,500 with a radius of 5 µm, 4.6 times of that (∼2800) offered by a conventional SWG [157]. By utilizing a trapezoidal-shaped SWG core, an enhanced sensing capability was analyzed and characterized by Yan et al., obtaining a high *Q*-factor of 9100, bulk sensitivity of 440.5 nm/RIU and surface sensitivity of 1 nm/nm with iDL of 3.9 × 10^−4^ [158]. To further improve the DL value, Huang et al. theoretically and experimentally optimized an SWG racetrack resonator in the TM mode to obtain a maximum *Q*-factor of 9800 and bulk sensitivity of 430 nm/RIU in water, which corresponds to a 32.5% improved iDL of 3.71 × 10^−4^ RIU compared to conventional TE-polarized SWG sensors [159]. Recently, Luan et al. developed two sensitivity enhanced SWG-based multi-box waveguide biosensors by merging slot and SWG structures, as presented in Figure 11d,e [162,163]. The expanded optical mode and the multiplied surface area for analyte interactions offer a highly improved light–matter contact at the sensor’s surface, thus resulting in a bulk sensitivity of 580 nm/RIU and surface sensitivity of ∼1900 pm/nm, respectively [162]. As shown in Figure 11c, SWG waveguides were also integrated into the MZI-based biosensor as the sensing arm by Sumi et al. in 2017. The device, with the sensing arm’s length of 100 µm, is designed to operate at an operating wavelength of 1550 nm in the TE mode with a length-dependent scalable sensitivity of 931 rad/RIU/mm [164].

#### 3.2.2. Vernier Effect Based Systems

**Figure 12:**
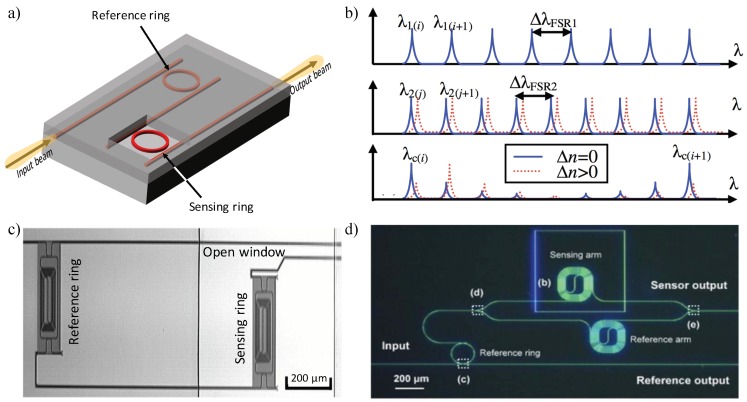
(**a**) Illustration of the Vernier effect sensing system consisting of two cascaded MRRs with different FSRs. The sensing ring is exposed to RI changes in its environment, while the reference ring is covered by the cladding. (**b**) Illustrations of calculated transmission spectra of the reference device ($\Delta \lambda_{\mathrm{FSR}1}$), sensing device ($\Delta \lambda_{\mathrm{FSR}2}$), and cascaded system, respectively. Red-dashed lines represent transmission spectra after an RI change above the sensing device, showing an amplified wavelength shift in the cascaded system. Figure adapted with permission from Reference [165] (**c**) Microscopic image of the two cascaded MRRs sensing device fabricated in SOI with an opening at the second MRR. Their footprint is reduced by folding the cavity. Figure adapted with permission from Reference [166]. (**d**) Microscopic image of the cascaded MZI and MRR sensor with an opening at the sensing arm of the MZI. Figure adapted with permission from Reference [167].

The Vernier effect is a method commonly used in calipers and barometers to enhance the accuracy of instrument measurements by overlapping two scales with different periods, of which one slides along the other one. The overlap between lines of the two scales is used to perform the measurement. Recently, Vernier-principle based sensors have been investigated in the SOI platform by cascading two or more optical devices with different FSR values, where one has the upper cladding removed and represents the RI sensor (as shown in Figure 12a). Due to the different FSRs between the sensing and reference (filter) devices, a spectral response with a major peak plus some minor peaks will be presented at the output. As shown in Figure 12b, the major peaks are located at the overlapped peaks of these devices, showing a Vernier FSR of the least common multiple of total FSR values, and the height of major peaks is determined by the amount of overlap. When the RI above the sensing device changes, the major peak shifts ($\Delta \lambda_{\max}$) discretely which equals to an integer multiple of the reference device’s FSR (Δ$\lambda_{\mathrm{FSR}}^{\mathrm{ref}}$), i.e., Δ$\lambda_{\max}$ = *m*Δ$\lambda_{\mathrm{FSR}}^{\mathrm{ref}}$ [165]. In this way, the Vernier effect cascaded sensor system yields an ultra-high sensitivity which is given by Dai [165]:

(24) $$S\;=\;\left(\lambda_{\mathrm{maj}}/n_{\mathrm{eff}}\right)\left[\frac{\Delta \lambda_{\mathrm{FSR}}^{\mathrm{ref}}}{\left(\Delta \lambda_{\mathrm{FSR}}^{\mathrm{ref}}-\Delta \lambda_{\mathrm{FSR}}^{\mathrm{sen}}\right)}\right]\;=\;MS_{0}$$

where $\lambda_{\mathrm{maj}}$ is the wavelength of the major peak, $\Delta \lambda_{\mathrm{FSR}}^{\mathrm{ref}}$ and $\Delta \lambda_{\mathrm{FSR}}^{\mathrm{sen}}$ are the FSRs of reference and sensing devices respectively, and S_0_ is the actual sensitivity of the single sensing device. Thus, the sensitivity of the optical sensor based on Vernier effect cascaded devices is *M* times improved than that of a single device, without requiring a narrow linewidth tunable light source or a high-resolution readout system. The trade off is that the readout is quantized, thus potentially limiting the minimum detection limits.

Earlier, the Vernier principle was applied to the design of integrated tunable lasers [168] and filters [169,170]. In 2009, Dai et al. proposed a sensing system that consists of two cascaded MRRs, theoretically showing a two orders higher sensitivity (on the order of 10^5^ nm/RIU) than that of a regular single-ring sensor due to the Vernier effect, and a DL highly related to the FSRs difference [165]. In parallel, He’s group pioneered in investigating cascaded MRR sensors according to the Vernier effect theoretically and experimentally in the TE [171] and TM [172] modes, yielding sensitivities of 1300 nm/RIU and 24,300 nm/RIU, respectively. In 2010, Claes et al. developed cascaded MRRs with very large roundtrip lengths presented in Figure 12c where FSRs difference is smaller than the FWHM of resonance peaks, and introduced a fitting procedure to reduce the smallest detectable wavelength shift, obtaining a experimental sensitivity as high as 2169 nm/RIU and DL, which is no longer limited by the $\Delta \lambda_{\mathrm{FSR}}^{\mathrm{ref}}$, of 8.3 × 10^−6^ RIU [166]. One year later, Hu et al. employed a suspended MRR for sensing by removing the SiO_2_ underneath, yielding a sensitivity up to 4.6 × 10^5^ nm/RIU and DL of 4.8 × 10^−6^ RIU [173]. In 2012, Passaro et al. introduced a Vernier effect sensing system for gas detection leveraging slot-waveguide based MRR as the sensing device; a sensitivity of the order of 10^5^ nm/RIU and DL as low as 10^−5^ RIU are achieved for detecting methane and ethane in the air [174]. Moreover, a three cascaded MRRs sensing system was reported in 2017 by Liu et al. with a high sensitivity of 5866 nm/RIU; the measurement range which used to be limited by the FSR of the sensing ring obtains a 24.7-fold increment compared with traditional cascaded MRRs [175].

The concept of sensitivity enhancement by employing MZIs to Vernier effect sensing systems was theoretically demonstrated by La Notte et al., by replacing the sensing MRR with a MZI. The proposed sensor is considered to reach an ultra-high sensitivity theoretically over 1000 µm/RIU and a very low DL of 10^−6^ RIU [176]. In 2014, Jiang et al. demonstrated an ultra-high sensitivity Si biosensor based on cascaded MZI and MRR with the Vernier effect (see Figure 12d). Experimental results indicate a sensitivity of 21,500 nm/RIU for MZI-ring sensor, 7.5 times higher than that (2870 nm/RIU) of a single MZI sensor [167].

### 3.3. Sensitivities Comparison

A sensor performance results comparison in the field of silicon photonic biosensors is presented in Table 1 along with different architectures as well as strategies to improve the S and DL values. Due to un-unified units of DL among different articles, bulk sensitivities in the unit of wavelength (or phase) shift per refractive index change are estimated from the results in the publications to serve as a comparison criterion. Moreover, other parameters and performance metrics such as light polarization and wavelength, system and intrinsic detection limits, and *Q*-factor are also presented.

### 3.4. Section Summary

From the experimental results presented in Table 1, bulk sensitivities are enhanced in sensing configurations applied by performance-improving strategies. However, their detection limit values show no growth but a downward trend for slot and SWG waveguide-based sensors. That matches well with the recently published work by Kita et al., who found out that sensor performance of slot and SWG waveguides are not truly better than strip waveguides for sensing [179]. By proposing a dimensionless figure of merit (FOM):

(25) $$FOM\;=\;\frac{\Gamma_{\mathrm{clad}}}{\alpha_{s}\times \lambda }$$

where $\Gamma_{\mathrm{clad}}$ is the optical confinement factor ($\Gamma_{\mathrm{clad}}\;=\;\frac{\partial n_{\mathrm{eff}}}{\partial n_{\mathrm{clad}}}$), and $\alpha_{s}$ is the scattering loss per unit length, both modal confinement and roughness scattering loss are taken into account for the comparison of various waveguide geometries by the authors. The model predicts that properly engineered TM-polarized strip waveguides claim the best performance compared to slot and SWG-based waveguides owing to their reduced propagation loss and longer accessible optical-path length [179]. Therefore, for the purpose of sensor performance enhancement (both the sensitivity and detection limit), more efforts are required to decrease the scattering loss for sidewall-roughness sensitive waveguides, such as slot and SWG geometries.

## 4. Label-Free Detection

Generally, two approaches for optical detection are employed by most biosensors: label-based detection and label-free detection. In labeled detection, a label is defined as an additional molecule that is chemically or temporarily attached to the immobilized target to enhance the quantitative signal. Examples include, but are not limited to, a dye molecule (chromophore), a fluorescent tag, or an enzyme. This labeling process can achieve an ultra-low DL (on the order of sub-parts-per-trillion) and provide additional specificity via secondary amplifications [26]. However, it requires sophisticated reagent selection and pairing, in addition to reagent modification including synthesis and purification, which potentially changes intrinsic properties of the capture probe and/or target molecules [180] and dramatically increases the cost and complexity of the assays. Moreover, due to the need for additional steps to perform label-based detection, it is ill-suited for real-time kinetic monitoring. To contrast, label-free detection has emerged as an appealing alternative to labeled detection, utilizing native molecular properties such as molecular weight (MW), RI, and molecular formal charge (FC) for target molecule monitoring. Label-free detection is not without its own drawbacks, as the method is only capable of providing sensitive and specific detection if non-specific binding (NSB) is low, or if the assay has sufficient controls to subtract the contribution of NSB. Additionally, label-free detection requires sufficient signal to be generated upon binding for the sensor to differentiate signal from noise; this can limit label-free detection for certain applications with especially low molecular weight target species, or targets that do not readily interact with specific capture probes/chemistries. Even with these limitations, a large number of biosensors designed for label-free detection have been investigated in the recent research literature [181,182,183], largely because the method greatly simplifies assays, can reduce both the time and number of steps required, and eliminates experimental uncertainty induced by the labeling process [184]. Additionally, label-free detection is highly amenable to the real-time kinetic evaluation of molecular binding and rapid quantification of analytes.

Since the first label-free optical biosensor was commercialized in 1990 by Biacore [10], an entire field has arisen developing new platforms for label-free biosensing, driven largely by the appeal of addressing the unmet need in medical diagnostics, biosensing, and environmental/biohazard/threat monitoring. Among the new transducers, optical devices based on the SOI platform are among the most promising. Their highly compact footprint, allowing simultaneous multiplexed detection on a single chip, and low fabrication cost in high volumes with CMOS-compatible processes, make them cheap enough to be considered fully disposable. Table 2 gives an overview of a wide variety of exemplary target analytes, arranged in descending molecular weight, that have been detected using label-free SOI-based biosensors, as well as their reported DLs. This survey demonstrates that SOI-based optical biosensors have a wide detection range for analytes with MWs on the order of kilodalton (kDa). For large molecules such as micrometer-sized cells and bacteria on the order of megadalton (MDa) or higher, their sizes may exceed the evanescent field range of the sensor and cause a invalid result. For small molecules (normally less than 500 Da), a detectable signal is difficult to achieve, especially for low concentrations, due to the low sensitivity or high noise level of SOI-based sensors.

## 5. Optical Sensing System Integration

To satisfy the need for system operations towards clinical and home healthcare diagnosis, integration is one of the key challenges to be solved [206]. The SOI platform is appealing since it offers the potential of optical component integration onto the same substrate. In recent years, massive amount of efforts have been made to integrate multiple functions to chip-scale silicon PICs, such as on-chip fluidic handling and optical analysis, as well as data processing [207]. These integrated sensing architectures show the ability for a high-density, lab-on-a-chip, and portable biosensing platform in the application of POC medical diagnosis. Here, we review research directed towards the integration of microfluidics, lasers, sensing devices and photodetectors (PDs) on Si substrates for biosensing applications.

### 5.1. Optofluidic Integration

Microfluidic systems have been regarded as an essential tool for modern biosensing research due to outstanding advantages such as low sample consumption, in-situ manipulation, short analysis time, controlled transportation, and high throughput [208,209]. Recently, a synergy technique called optofluidics has emerged, which integrates microfluidics and photonic architectures to enhance each entity’s function and performance [210]. Introducing optofluidics to silicon photonic biosensing systems not only combines fluid and light for improved sensing capability and simplification of microsystems but satisfies the function of on-chip, label-free, real-time detections. In addition, optofluidic sensors are extremely suitable for evanescent field RI detection, since the change of RI scales with the analyte bulk concentration or surface density, rather than the number of molecules in total [210].

Polydimethylsiloxane (PDMS) has become the most popular material in the academic microfluidics community since it is inexpensive, easy to fabricate, flexible, optically transparent, and biocompatible [211]. More importantly, PDMS material can be permanently bound to SiO_2_ substrates after oxygen plasma treatment [212], which provides a simple and fast approach to build leakage-free microfluidic channels on SOI-based sensors. Many silicon photonic devices including MZIs [102,193], MRRs [162] and PhCs [104,204,209] have employed PDMS microfluidic systems mounted on top as a convenient optofluidic delivery method for analyte detection. However, PDMS also shows some drawbacks. On the one hand, PDMS is not suitable for the integration or deposition of electrodes directly on the surface, and has problems such as adsorption of small hydrophobic molecules, swelling in organic solvents, water permeability, and incompatibility under very high-pressure operations [211]. On the other hand, due to the irreversible bonding process, chips are not reusable after mounting the PDMS microfluidic block, and most of the area on the chip only serves as a mechanical support for the fluidic inlet and outlet but not for sensing, which negatively impacts the unit cost [213].

Another commercially available material, negative tone photoresist SU-8, has been employed for on-chip optofluidics recently. SU-8 was originally developed as a high-resolution photoresist for the microelectronics industry. Because of its transparency in the near-infrared spectrum and biocompatibility, a thin layer of SU-8 coating with microfluidic patterns has been investigated on silicon photonic biosensing systems [45,213], which improves the alignment precision compared to PDMS microfluidics bonding. Furthermore, SU-8 can also be used as a cover material for interface passivation of on-chip electrical connections due to its high-resolution patterning and insulation abilities. However, the manufacturing process of the SU-8 microfluid requires the use of clean room facility equipment involving complex and numerous processing steps, which hinders mass production at a low price. In addition, variation in conditions such as humidity and SU-8 composition may affect fabrication protocols, contributing to batch-to-batch variability [214]. Other materials such as glass [215], polycarbonate (PC) [216], cyclic olefin copolymer (COC) [217] and epoxy [218] were also reported for the on-chip optofluidic integration.

Digital microfluidics is an emerging technology in the field of biosensing by using microdroplets instead of continuous flows. Microliter or picoliter drops can be generated, transported, mixed, and split in miniaturized reaction chambers without moving equipment such as pumps or valves, which offers great potential for pump-free high-throughput liquid handling and avoids on-chip cross-contaminations [219]. Electrowetting is the most commonly used technique for microdroplet actuation, which refers to electric field-induced interfacial tension changes between the liquid and the dielectric layer, resulting in a contact angle change, and thus droplet movement [220]. The integration of SOI-based optical sensors and digital microfluidics has been demonstrated by utilizing MRRs [219,221] and microdisks [222] since 2008, showing comparable sensitivities to their counterparts measured in standard optofluidic systems. Another approach for eliminating pumps and valves has been investigated recently by employing an integrated, microtechnological pumping method. The actuation principle is mainly based on the deflection of a deformable polymer membrane to push the liquid from the reservoir towards the microfluidic channel, where the deflection results from the increased pressure underneath the membrane by the electrolytically generated gas [223]. Geidel et al. showed an integrated microfluidic design consisting of multiple reservoirs and electrochemical pumps for time-controlled delivery, which has been tested and validated by SiN-based MRR biosensors, indicating the possibility of on-chip liquid handling integration for high-level miniaturized optical biosensors [216]. However, the prototype worked with a low sensitivity due to the unselective binding within the cartridge or selective binding exceeding the evanescent field on the MRR, which requires further optimizations for the surface biofunctionalization.

### 5.2. Optoelectronic Integration

One of the biggest roadblocks towards the large-scale commercialization of photonic biosensors is the low-cost high-yield integration of light sources to operate reliably whilst consuming minimal power. These goals are usually traded-off against each other with the choice of platform for integrating the light source, the sensor device, and the photodetector (PD) to achieve a complete lab-on-a-chip system. For instance, to benefit from a high-yield and low-cost production, leveraging existing CMOS fabs seems to be the ideal solution. This requires the integration of these three elements on a single Si CMOS-compatible die. However, integrating the active laser source with the passive sensor device and the PD remains a challenge. Several techniques utilized for the chip-scale optoelectronic integration are presented below, and advantages brought as well as challenges faced by each method are highlighted.

#### 5.2.1. On-Chip Lasers

Driven by the promises lasers on Si hold for optical communication [224], several groups across the world have demonstrated integrated lasers on Si dies implemented using either group IV materials (Si or Ge) or group III/V compounds [225]. While using group IV elements seem to be an appealing and practical solution in terms of cost and portability, existing methods using Si cannot yet render an electrical I/O-based lab-on-a-chip because they rely on optical pumping mechanisms [226,227], making it an unattractive solution at the moment. Electrically-pumped Ge lasers integrated on Si, however, have been demonstrated [228]. Despite its indirect bandgap, straining and n-doping Ge can tailor its bandgap to make it direct [229]. Repercussions of this approach are high threshold currents [228] thus increasing the total power budget of the biosensors.

On the other hand, III/V lasers integrated on Si have been demonstrated with a much higher efficiency in comparison to Ge, thanks to their direct bandgap and superior gain characteristics. While monolithic integration of III/V compounds on Si seems to be the optimum solution for ease of portability and highest density integration, the biggest bottleneck towards the direct monolithic growth of III/V compounds on Si lies in the lattice and thermal expansion coefficient mismatch between the Si material and III/V compounds [225]. To solve this problem, three main approaches have been demonstrated to integrate III/V lasers on Si chips: (i) direct mounting; (ii) hybrid approaches through direct and indirect bonding heterogeneous integration; and (iii) monolithic integration using sophisticated growth techniques.

Direct mounting includes flip-chip bonding using solder bumps through edge-coupled III/V to Si waveguides [230,231,232] or through vertical coupling using SiO_2_-SiO_2_ bonding techniques [233,234]. The main advantage this method brings is the independent growth of III/V materials on its native substrate, thus benefiting from the merits of a III/V compound as a gain medium. In addition, the solder bumps provide a means to dissipate the generated heat from the III/V die to the Si substrate leveraging its high thermal conductivity [235]. Furthermore, with a rigorous design of spot-size convertors and accurate alignment, high wall-plug efficiency (WPE, the ratio of the output optical to input electrical power), up to 35% [236] can be achieved. The laser’s cavity can be shared between the III/V gain chip and Si, known as external cavity lasers (ECLs). ECLs allow for the independent control over the laser’s properties such as the linewidth [237], wavelength tuning [238], and stabilization using on-Si chip electrical control [73,239,240]. Nevertheless, common issues of direct mounting integration include low efficient end-coupling between the III/V and Si waveguides requiring precise alignment, and degradation in the laser’s overall performance due to possible back reflections into the laser source [241]. Even if aligned at the microscale, the process is both costly and tedious [235] which adds to the overall cost of a lab-on-a-chip system making it an expensive solution and limiting its usage for prototyping purposes.

An alternative and more efficient way of integrating III/V gain materials on Si substrates is through indirect (using metal or polymer layers) or direct bonding techniques [225,242], commonly referred to as hybrid or heterogeneous integration. The biggest advantage of heterogeneous integration above direct mounting is that it does not require the precise alignment at the microscale, since the III/V active layers are lithographically aligned with high precision. Direct bonding can be achieved using Oxygen plasma at low temperatures. This was first demonstrated by Bowers et al. [243,244], and due to it being a cost-effective solution, this work resulted in a startup, Aurrion Inc. that was later acquired by Juniper Networks [245,246]. Direct bonding has the advantage of not requiring the addition of any extra layers, and lasers formed this way can achieve low threshold currents [225]. Indirect bonding, on the other hand, was demonstrated using metal-assisted adhesive bonding [247,248,249], whereas others have used polymers such as divinylsiloxane-bis-benzocyclobutene (DVS-BCB) [242,250,251]. While metals provide better heat dissipation due to their high thermal conductivity, polymers are more straightforward to fabricate and, unlike metals, do not absorb light. Polymers, however, have the disadvantage of having a high thermal resistance thus localizing heat. To mitigate its effects, Roelkens has fabricated polymers with <50 nm thickness, thus reducing its effect in localizing the heat [242]. The same group have extended this technique and demonstrated light sources at a various wavelength for biosensing applications [252]. This makes heterogeneous integration a scalable technique that enables dense integration of III/V in SOI platforms, thus reducing the potential costs of a lab-on-a-chip system. Furthermore, ECLs can be implemented in the hybrid approach, thus leveraging the merits that ECLs brings [238].

There are several monolithic approaches for integrating III/V lasers and active devices on Si substrates. Epitaxial layer overgrowth (ELOG) is one way to overcome the formation of threading dislocations that arise due to the lattice and thermal expansion mismatch between III/V and Si materials [253]. The process is yet more complicated in comparison to the formerly mentioned techniques.

**Figure 13:**
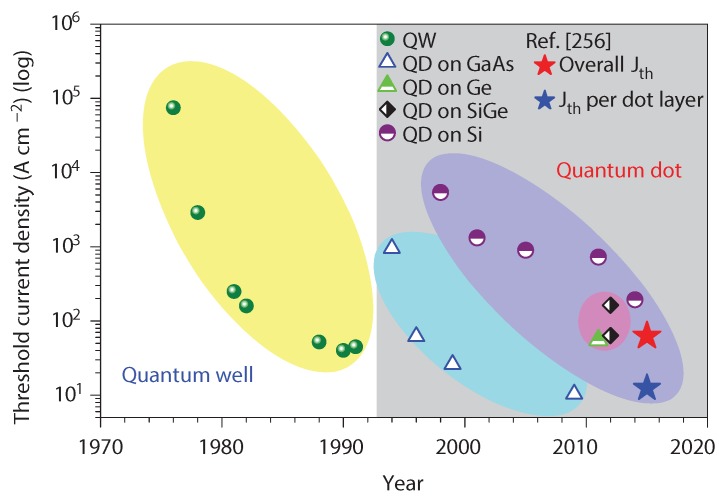
Historical development of low-dimensional heterostructure lasers, showing the record threshold current densities. The blue and red stars indicate the threshold values achieved in Reference [263] for a single and multiple quantum dot (QD) layers, respectively. Figure adapted with permission from Reference [263].

While the choice of III/V integration method on Si directly influence the overall laser’s performance, the choice of the III/V active gain medium physical structure is equally important. For instance, to achieve a low-power and reliable (avoiding overheating) operation, the WPE of the laser should be maximized. The WPE or the conversion efficiency is a crucial figure of merit in a laser design, which is dependent upon the threshold current, electron density and the internal losses in the laser’s cavity. These parameters are dependent upon the band structure of the chosen active gain medium, which is engineered by physically restricting the electrons motion to form double heterostructure (DH), quantum well (QW), quantum wire (QWR) or quantum dot (QD) structures. Among the various structures reported, QDs stand out as they offer superior properties compared to their counterparts DHs, QWs, or QWRs as shown in Figure 13. Thanks to the tight electron confinement, thus increasing the optical gain dependence on the current density, which reduces the transparency current and makes the threshold current density temperature insensitive [254]. Motivated by lowering the threshold current and making a temperature insensitive laser, Dingle and Henry proposed the QD laser back in 1976 [255]. Since its analysis by Arakawa and Sakaki in 1982 [256], a plethora of applications on-Si platform has leveraged the merits QD lasers brought [257,258,259,260]. Perhaps, one of the main reasons behind the proliferation of QDs lies in its minimal sensitivity to defects [261], which drew increased attention and allowed for the growth of III/V QDs on Si [262]. This is very promising; however, its compatibility with CMOS processes remains controversial [73]. Recently, researchers at University College London [263] demonstrated electrically pumped III/V QD lasers on Si with superior characteristics, such as a low threshold current density of 62.5 A/cm^2^, room temperature output power of >105 mW, and over several months of reliable continuous operation, giving an estimated failure of over ten years of operation. This holds great promises towards the high-volume practical realization of low-cost photonic biosensors.

#### 5.2.2. On-Chip Detectors

For a lab-on-a-chip system with electrical I/Os, an on-chip photodetector is required to convert the light signal for further processing. There are several on-Si PDs implemented either using III/V compounds, or using group IV elements such as Si or Ge. The choice of PDs depends on the detection wavelength of interest. Wang et al. have heterogeneously integrated III/V PDs on Si substrate for operation at a wavelength of 2 µm [252]. Other techniques explored include thermo-electric PDs [252,264]. However, across the C-band, besides III/V compounds [242], Ge and Si could be used for photodetection. The main advantages of using Si or Ge is their ease of fabrication with a CMOS fab. Despite Si’s transparency at the C-band, doping Si can increase the Si waveguide’s sensitivity to incoming light across the C-band either due to surface states [265], or due to the introduction of mid-band-gap defect states [265,266,267,268]. Si-based defect-mediated PDs, however, suffer from either low responsivities or large photoconductive gain at the expense of a much larger dark current [267], which is undesirable for biosensing applications. Ge-based PDs, however, have superior characteristics. Recent results showed Ge on Si PDs with a high responsivity of 0.74 A/W and low dark currents of less than 4 nA [269]. Their integration into an on-chip biosensor was also demonstrated in Reference [213], and its performance was analyzed. These characteristics make Ge-based PDs ideal for biosensing at a wavelength of 1.3 µm or 1.5 µm in the SOI platform.

### 5.3. Readout

For conventional evanescent field biosensing techniques, two aforementioned methods are usually employed for the quantitative detection of analytes at the sensor’s surface in real-time: the first one is monitoring the wavelength (or phase) shift in the transmission spectrum through scanning the input light source wavelength, which allows a large dynamic range for sensors; the other one is detecting the transmission intensity change caused by shifts at a fixed wavelength and providing precise detection with a very small concentration of analytes [270,271]. Both spectral domain approaches require precise optical spectrum scanning and processing systems, such as a wavelength-tunable laser, high-resolution photodetector or optical spectrum analyzer. Correspondingly, two types of spectral noise sources, wavelength noise and intensity noise, are categorized: wavelength noise ($\sigma_{\mathrm{wavelength}}$) is mainly generated from the light source wavelength shift and thermally influenced fluctuations of the sensor, whereas intensity noise ($\sigma_{\mathrm{intensity}}$) is caused by light source intensity fluctuations, the variation of input coupling, and PD noise [272]. Another important factor, the spectral resolution ($\sigma_{\mathrm{resolution}}$) of the system setup, can also limit the precision of the spectral location, which highly depends on the measurement setup, i.e., the laser or the optical readout. Therefore, the total noise variance in the sensing system can be approximated by summing all the individual noise variances [57]:

(26) $$3\sigma \;=\;3\sqrt{\sigma_{\mathrm{wavelength}}^{2}+\sigma_{\mathrm{intensity}}^{2}+\sigma_{\mathrm{resolution}}^{2}}.$$

Several approaches can be applied to improve the system noise for silicon photonic sensors. As mentioned before, *Q*-factor plays an important role in determining the DL of a sensor. That is because having a high *Q*-factor (narrow FWHM) can filter the spectral noise effectively and lead to a low spectral deviation from the actual extremum [57]. Another one is introducing optical spectrum curve fitting, which is a powerful tool to enhance the spectral resolution. Taking into account of the entire spectrum, a fitting process can improve the eventual signal-to-noise ratio (SNR) by $\sqrt{N}$, where *N* is the total number of data points in the spectrum [29]. By applying this algorithm to silicon photonic biosensors, a wavelength measurement precision much smaller than both the light source linewidth and the peak FWHM is achieved [272], with a factor of approximately 10–10^3^ [59]. Therefore, the system DL with an improved linewidth in the spectrum readout can greatly enhance sensor performance as compared to the intrinsic DL using the peak linewidth according to Equations (5) and (12).

Recently, Wang et al. proposed a biosensing scheme using a coupled-resonator optical-waveguide (CROW) in the SOI platform, where a series of coupled MRRs cause a specific spatial domain scattering pattern by applying a fixed wavelength to excites the CROW [270]. Based on the captured intensity of the light-scattering of each MRR, the whole structure intensity pattern dependent on the RI change above the CROW is presented as the readout scheme by the imaging camera. By introducing different concentrations of NaCl solutions to an 8-MRR CROW sensor, a bulk sensitivity of ∼752 RIU^−1^ and DL of ∼6 × 10^−6^ RIU are achieved [270]. Although no spectrum scanning system is needed in this design for the sensor’s excitation and detection, the simultaneous imaging system still impedes the goal of the low cost, portable development.

### 5.4. State-Of-The-Art CMOS-Chip Packaging

Compared to traditional benchtop sensors and instrumentation, biosensors that rely on CMOS processes offer lower cost, lower power and smaller size with a high-density on-chip sensing array [273]. In terms of lab-on-a-chip monitoring, the primary challenge is the integration of sensing arrays interfaced with fluid samples and electrical interconnects for data processing on CMOS substrates. Furthermore, die-level CMOS substrates are always millimeter-sized which obstructs the on-chip microfluidics and electrical interconnections integration for high-throughput.

**Figure 14:**
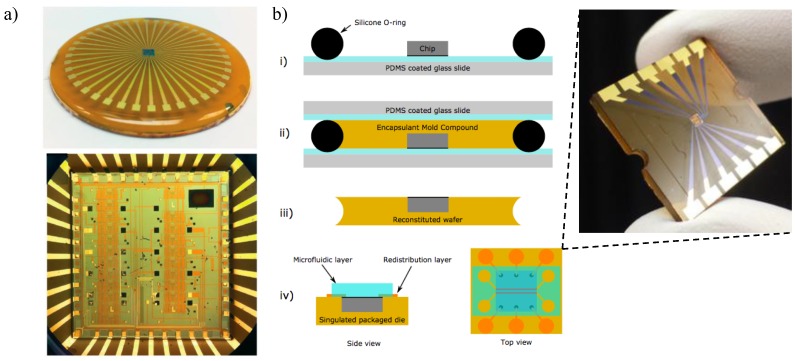
(**a**) Images of the die-embedded epoxy handle wafer with thin-film Au connections fan-out from the chip to the edge, and close-up view around the die. Figure adapted with permission from Reference [276]. (**b**) Schematic of lab-scale Fan-Out Wafer-Level-Packaging (FOWLP), and image of the 16 × 16 mm^2^ packaged CMOS die after singulation. Figure adapted with permission from Reference [213].

To overcome these difficulties, several post-CMOS approaches have been investigated as system-level packaging to implement electronic and biological detection functions. Fluid barrier materials, such as PDMS, epoxy, SU-8, oxide/nitride, and parylene, have been employed for integrating CMOS chips with microfluidics. Li et al. reported a chip-in-package process utilizing wire bonding technology for the die-level on-CMOS biosensor integration [274]. By depositing a 2-µm-thick parylene layer as the insulating coating, the biosensor is enabled for operations in liquid with a good functionality of CMOS electronics [274]. Huang et al. developed a lab-on-CMOS platform for electrochemical microsystems by using oxide/nitride/oxide (ONO) passivation layers, which allows the functional integrity of multi-channel microfluidic structures and on-CMOS electrodes [275]. For the size disparity between the CMOS chip and on-chip microfluidics, die-level CMOS chips have been encapsulated into a substrate carrier which enlarges the surface area for further processes. In 2014, Datta-Chaudhuri et al. presented a simple packaging method for die-level CMOS foundry-fabricated chips, which are embedded in epoxy handle wafer for a level, enlarged surface, allowing subsequent post-processing and microfluidic integration [276]. Parylene-C was selectively exposed to the surface for the passivation of electrical connections. As shown in Figure 14a, due to the flat surface around the chip, good electrical continuity of fan-out metal traces from the chip to the edge of the wafer is achieved, enabling the subsequent off-chip data communication [276]. Similar approaches have been considered for PICs. Laplatine et al. developed a novel system-level architecture by embedding the individual photonic-electronic die into a two-inch epoxy wafer, with electrical interconnects and microfluidic channels based on a lab-scale Fan-Out Wafer-Level-Packaging process (FOWLP) presented in Figure 14b [213]. SU-8 was selected for the microfluidic channels patterning as well as electrical connections passivation. By characterizing on-chip Ge PD components in the photovoltaic mode, they demonstrated an approach for biomolecule detections even with a low optical power [213]. In addition, sensor performance was also characterized by introducing standard NaCl solutions and bio-sandwich assays to FOWLP-packaged chips. A bulk sensitivity of 220 nm/RIU is achieved, close to the sensing capability of the passive counterpart [277]. Similarly, a CMOS-compatible epoxy chip-in-carrier process was developed by Lin et al. [278]. By introducing a planar screen-printed silver ink metallization technique with mounted multichannel PDMS microfluidics on the device’s surface, electrochemical and microfluidic experiments were evaluated by interconnect resistance measurements, showing high effectiveness for lab-on-CMOS applications to achieve desired capability with high yield and low material and tool cost [278].

## 6. Conclusions

Over the past two decades, silicon photonics technology has attracted enormous attention and research effort in optoelectronic integration to impact multiple application areas. Leveraging the mature CMOS manufacturing technology, Si-based optical biosensing platforms have experienced huge breakthroughs in chip-scale integration and miniaturization for hand-held, label-free bio-diagnosis with high-volume production at low cost. By monitoring perturbations of the guided light in the waveguide, target molecules that change the RI in the vicinity of the sensor can be detected in real-time, showing a significant sensing capability down to sub-femtomolar. Moreover, some of the Si-based biosensing architectures have even been commercialized for label-free detection by companies such as Axela Inc., Corning Inc., and Genalyte Inc., through employing optical gratings, microplates, and microresonators into the sensing platform. However, due to the challenge of the monolithic integration on Si substrate, achieving a complete chip-scale integration of the portable biosensing platform for POC diagnosis requires further development. Compared to very commercially-mature label-free biosensing technique, i.e., SPR, the Si-based sensing approach still needs improvement in sensitivity for label-free detection of small molecule analytes to fulfill the market demand. Thanks to the intensive research effort throughout the world, we firmly believe that true lab-on-a-chip, portable biosensing devices will be realized and revolutionize global healthcare.

## Figures and Tables

**Table 1:** Performance metrics comparison of selected optical biosensors (WG, waveguide; Wvl, wavelength, 1.55 µm where not specified).

Sensor Type	Sensor Configuration	Strategy	Optical Mode	*Q*-Factor (×10^3^)	Bulk Sensitivity (RIU^−1^)	System Detection Limit (RIU)	Intrinsic Detection Limit (RIU)
**Interferometer**	MZI	Vernier	TE	N/A	2.15 × 10^4^ nm	N/A	N/A [167]
		Suspended	TE	N/A	740 nm	N/A	4 × 10^−5^ [146]
		Slot	TE	N/A	1730 × 2π rad	1.29 × 10^−5^	N/A [134]
		1.31 µm Wvl	TE	N/A	540 × 2π rad	N/A	N/A [148]
		N/A	TM	N/A	460 × 2π rad	3.3 × 10^−5^	N/A [17]
		N/A	TE	N/A	300 × 2π rad	N/A	N/A [44]
**Microcavity**	Ring	Vernier/suspended	TM	N/A	4.6 × 10^5^ nm	N/A	4.8 × 10^−6^ [173]
		Vernier	TM	15	2.43 × 10^4^ nm	N/A	N/A [172]
		Vernier	TE	20	1.3 × 10^3^ nm	5.05 × 10^−4^	N/A [171]
		Slot/critical coupling	TE	6	1.3 × 10^3^ nm	N/A	<10^−4^ [130]
		Multi-box SWG	TE	2.6	580 nm	N/A	1.02 × 10^−3^ [162]
		SWG	TE	7	490 nm	2 × 10^−6^	5.5 × 10^−4^ [161]
		SWG	TM	9.8	429 nm	N/A	3.71 × 10^−4^ [159]
		Slot	TE	0.33	298 nm	4.2 × 10^−5^	1.59 × 10^−2^ [128]
		Suspended	TM	12	290 nm	N/A	N/A [145]
		Thin WG	TM	4.5	270 nm	N/A	1.2 × 10^−3^ [19]
		N/A	TM	10.1	200 nm	N/A	7.5 × 10^−4^ [19]
		Thin WG	TE	24	133 nm	N/A	5 × 10^−4^ [142]
		1.31 µm Wvl	TM	33.5	113 nm	N/A	1.49 × 10^−3^ [30]
		1.31 µm Wvl	TE	9.8	91 nm	N/A	3.5 × 10^−4^ [30]
		N/A	TE	15	38 nm	N/A	2.7 × 10^−3^ [30]
	Disk	N/A	TM	16	142 nm	N/A	6.8 × 10^−4^ [21]
		Suspended	TM	0.1	130 nm	8 × 10^−4^	1.18 × 10^−1^ [144]
		N/A	TE	33	26 nm	N/A	1.8 × 10^−3^ [21]
**Photonic crystal**	2D	Slot	TE	50	1.5 × 10^3^ nm	7.8 × 10^−6^	2.07 × 10^−5^ [138]
		N/A	TE	0.4	200 nm	2 × 10^−3^	1.88 × 10^−2^ [86]
		Ring-slot	TE	11.5	160 nm	N/A	8.75 × 10^−5^ [177]
	1D	Slot	TE	174	815 nm	N/A	1 × 10^−5^ [178]
		N/A	TE	3	130 nm	7 × 10^−5^	4 × 10^−3^ [104]
**Bragg grating**	Phase-shifted	Multi-box SWG	TE	6.2	610 nm	N/A	4 × 10^−4^ [163]
		Slot	TE	15	340 nm	N/A	3 × 10^−4^ [135]
		1.31 µm Wvl	TM	76	106 nm	N/A	1.6 × 10^−4^ [30]
		N/A	TE	27.6	59 nm	N/A	9.3 × 10^−4^ [115]
	Uniform	N/A	TE	N/A	182 nm	N/A	N/A [22]

**Table 2:** Overview of selected biomolecules that have been detected by optical sensors using label-free method (CFU, colony-forming unit; HAU, hemagglutination unit; VP, viral particle).

Biological Material	Target	Weight	Sensor Type	Waveguide Material	Detection Limit
**Cell**	*E. coli* O157:H7	1 pg	MRR	Hydex	10^5^ CFU/mL [68]
			MRR	Si	10^8^ CFU/mL [185]
**Virus**	Avian influenza virus	542 MDa	MZI	Si_3_N_4_	5 × 10^−4^ HAU/mL [186]
	Herpes simplex virus	96 MDa	YI	Si_3_N_4_	850 VP/mL [187]
	Bean pod mottle virus	7 MDa	MRR	Si	1.43 pM [118]
	Human papillomavirus	5 MDa	PhC	Si	1.4 nM [188]
**Protein**	Immunoglobulin G	150 kDa	PhC	Si	1 ng/mm^2^ [85]
			MZI	Polymer	3.1 nM [148]
			Vernier MRR	Si	47.3 nM [189]
	(Strept)avidin	55-68 kDa	MZI	SiO_x_N_y_	2.14π/nm [190]
			PhC	Si	2.5 fg [25]
			PhC	Si	344 pm/nm [191]
			Slot MZI	Si_3_N_4_	18 fM [140]
			PhC	Si	49 fM [192]
			MRR	Si	60 fM [72]
			MRR-MZI	Si	20 pM [193]
			MRR	SiO_2_/Si_x_N_y_	0.1 nM [62]
			MRR	Si	0.15 nM [18]
			Slot disk	SiN_x_	0.55 nM [132]
	Human serum albumins	67 kDa	YI	Si_3_N_4_	20 fg/mm^2^ [194]
			MRR	Si	3.4 pg/mm^2^ [195]
	Prostate specific antigen	28 kDa	MRR	Si	0.4 nM [196]
			Slot MRR	SiN	1.79 nM [129]
	C-reactive protein	25 kDa	MZI	Si_x_N_y_	84 fM [197]
			MRR	Si	0.4 nM [121]
			MZI	SiN	0.78 nM [198]
**Nucleic acid**	RNA	7–40 kDa	MRR	Si	53 fM [199]
			MRR	Si	150 fM [200]
			Slot MZI	Si_3_N_4_	1 nM [201]
	DNA	7–12 kDa	MZI	Si_3_N_4_	300 pM [202]
			Slot MZI	Si_3_N_4_	1 nM [140]
			MRR	Si	1.95 nM [119]
			PhC	Si	19.8 nM [203]
			MRR	Hydex	100 nM [68]
**Small molecule**	Gentamicin	478 Da	PhC	Si	0.1 nM [204]
	biphenyl-4-thiol	186 Da	PhC	Si_3_N_4_	N/A [205]
