# Erratum: Luan, E.X.; Shoman, H.; Ratner, D.M.; Cheung, K.C.; Chrostowski, L. Silicon Photonic Biosensors Using Label-Free Detection. *Sensors* 2018, *18*, 3519

**DOI:** 10.3390/s19051161

**Published:** 2019-03-07

**Authors:** Enxiao Luan, Hossam Shoman, Daniel M. Ratner, Karen C. Cheung, Lukas Chrostowski

**Affiliations:** 1 Department of Electrical and Computer Engineering, University of British Columbia, 2329 West Mall, Vancouver, BC V6T 1Z4, Canada; hoshoman@ece.ubc.ca (H.S.); kcheung@ece.ubc.ca (K.C.C.); lukasc@ece.ubc.ca (L.C.); 2 Department of Bioengineering, University of Washington, 3720 15th Ave. NE, Seattle, WA 98195-5061, USA; dratner@uw.edu

The authors wish to make the following corrections in their published paper in Sensors [1].

In the last paragraph of page 8, the original article printed “Later, in 2010, Skivesen et al. achieved an improved DL of 6.75 × 10^−4^ RIU by tracking sharp fringes appearing in the slow-light regime near the edge of the guided band.” The last name of the first author and the detection limit value are incorrect. It should be revised as follows: “Later, in 2010, García-Rupérez et al. achieved an improved DL of 3.5 × 10^−6^ RIU by tracking sharp fringes appearing in the slow-light regime near the edge of the guided band.”

In the reference section, reference number 116 was incorrect. It was printed as “Joannopoulos, J.D.; Johnson, S.G.; Winn, J.N.; Meade, R.D. Photonic Crystals: Molding the Flow of Light; Princeton University Press: Princeton, NJ, USA, 2011.” It should be revised as follows: “Popović, M. Theory and design of high-index-contrast microphotonic circuits. PhD Thesis, Massachusetts Institute of Technology, Cambridge, MA, USA, 2008.”

Reference number 193 was incorrect. It was printed as “Martincek, I.; Kacik, D. A PDMS microfiber Mach–Zehnder interferometer and determination of nanometer displacements. *Opt. Fiber Technol.* **2018**, *40*, 13–17.” It should be revised as follows: “Yoshida, S.; Ishihara, S.; Arakawa, T.; Kokubun, Y. Highly sensitive optical biosensor based on silicon-microring-resonator-loaded Mach–Zehnder interferometer. *Jpn J. Appl. Phys.* **2017**, *56*, 04CH08.”

The authors would like to sincerely apologize for any inconvenience caused to the readers by these changes. References
